# Prenatal Stress Rewires the Gut–Brain Axis: Long-Term, Sex-Specific Effects on Microbiota, Intestinal Barrier, and Hippocampal Inflammation †

**DOI:** 10.3390/nu17172812

**Published:** 2025-08-29

**Authors:** Floriana De Cillis, Giulia Petrillo, Ilari D’Aprile, Moira Marizzoni, Samantha Saleri, Monica Mazzelli, Valentina Zonca, Maria Grazia Di Benedetto, Marco Andrea Riva, Annamaria Cattaneo

**Affiliations:** 1 Department of Pharmacological and Biomolecular Sciences, University of Milan, 20133 Milan, Italy; 2 Biological Psychiatry Laboratory, IRCCS Istituto Centro San Giovanni di Dio Fatebenefratelli, 25125 Brescia, Italy

**Keywords:** early-life stress, gut–brain axis, gut microbiota, intestinal permeability, resilience, vulnerability, behavioural alterations, hippocampus, inflammation, microglia

## Abstract

**Background:** The gut microbiota and the gut epithelium play a central role in maintaining systemic and brain homeostasis from early life. Stressful experiences during sensitive developmental windows can disrupt this balance, increasing long-term susceptibility to psychiatric disorders. However, the mechanisms through which early-life alterations in the microbiota influence brain development and function remain poorly understood. Here, the sex-specific impact of prenatal stress (PNS) on gut integrity and microbial composition in adult offspring was explored. **Methods:** Thirty dams were mated and randomly assigned to PNS or control. Offspring microbiota was analysed through 16S rRNA sequencing, intestinal morphology with morphometric analyses, and tight junctions using qPCR and immunofluorescence. **Results:** Exposure to PNS was associated with reduced intestinal surface area in males and shortened crypts in females. In both sexes, PNS caused a decrease in the expression of ZO-1, suggesting impaired gut barrier integrity. 16S rRNA sequencing revealed, furthermore, that PNS exposure was associated with a decrease in beneficial genera, including *Akkermansia* in males and *Clostridia vadinBB60* in females, along with an increase in the pro-inflammatory genus *Anaerotruncus*, regardless of sex. Notably, some of these alterations were more pronounced in PNS-exposed animals that showed impaired sociability, highlighting gut microbiota inter-individual variability in the response to early-life adversity. Moreover, selected microbial changes show significant correlations with the behavioural outcomes, as well as with intestinal morphology or brain inflammatory markers. **Conclusions:** Together, these findings pinpoint the gut as a central player in stress vulnerability and highlight specific microbial signatures as promising biomarkers and therapeutic targets for stress-related disorders.

## 1. Introduction

Mental health disorders are recognised as one of the leading causes of disability worldwide. Among the risk factors shaping vulnerability to this condition, exposure to stressful events early in life (ELS) has been identified as a major contributor [1]. Indeed, during the first years of life, the brain of newborns is more sensitive to environmental distress, which could trigger long-term consequences on different domains such as cognition, emotion and sociability [2,3,4].

Epidemiological and preclinical studies largely support this association and have also highlighted the hypothalamic–pituitary–adrenal (HPA) axis and the immune responses [5,6] as the main biological systems underlying this association [7,8]. Moreover, changes in the gut microbiome have also been widely investigated as possible mediators of the effects of adverse environments on brain functioning and behaviour [9]. The gut microbiome is a complex ecosystem consisting of trillions of microorganisms including bacteria, viruses, protozoa, fungi, and archaea that colonise the intestine immediately after birth [10,11]. From the earliest years of life, microbial commensals play a crucial role in several bodily functions, including the development of the endocrine and immune systems [12,13], and the growth and maintenance of the gut barrier [14].

Negative perinatal events, such as stress exposure, can disrupt maternal behaviour and affect several biological systems, including maternal gut microbiota composition [15,16]. These changes may, in turn, influence the physiological development of the offspring’s microbiome, contributing to dysbiosis and an inflammatory state within the gut [17,18,19]. Importantly, through the gut–brain axis, such microbiome changes can modulate brain mechanisms, cognitive functions, and behaviour [20], with the hippocampus representing a key region in this communication, particularly under stress exposure [21,22].

In this context, particular attention has recently been directed toward crypt-associated microbiota, microbial communities residing near to intestinal crypts and the underlying immune system [23,24]. Due to their strategic location, these microbial populations may play a pivotal role in regulating local immune responses and maintaining gut homeostasis.

Within this framework, it has been shown that prenatal stress (PNS) can lead to long-lasting immune alterations in the gut. Specifically, adult offspring exposed to PNS exhibit increased expression of pro-inflammatory cytokines such as Interleukin-1 beta (IL-1β), Interleukin-6 (IL-6), and Tumor necrosis factor alpha (TNFα) in the colon, together with elevated intestinal permeability, as indicated by decreased expression of tight junction (TJ) proteins and increased absorption of fluorescein isothiocyanate-dextran [25]. This increased gut permeability, often referred to as a “leaky gut”, can facilitate the translocation of microbial products and inflammatory mediators into the systemic circulation [26], which, under dysbiotic conditions, may allow pathogenic bacteria and their metabolites to reach the brain, thereby contributing to neuroinflammation and behavioural disturbances [27,28].

Supporting this, Gur and colleagues reported that adult offspring of stressed dams not only exhibited altered gut microbiota but also displayed reduced social behaviours and signs of neuroinflammation [29]. Similarly, our research group have recently demonstrated that exposure of rats to PNS leads to increased inflammation in the ventral hippocampus (VH) of adult animals, which is associated with impaired sociability [30]. Interesting, these alterations did not emerge uniformly across all exposed animals, highlighting individual variability in stress responsivity.

Despite the evidence that the gut microbiota could represent a key mediator of the long-term effects of ELS on brain function, the molecular mechanisms linking microbiota alterations to brain inflammation remain poorly understood. Building on the previous conference abstract [31], the present study aims to investigate how PNS shapes gut microbial composition and functions and how these changes may contribute to neuroinflammation and behavioural alterations through gut–brain axis pathways. To address this aim, a preclinical model of PNS exposure was employed to dissect the complex mechanisms underlying gut–brain communication. Animal models allow the analysis of specific variables and features of the brain and the gut that are not easily accessible in human populations. Moreover, animal studies enable not only the identification of associations, as in humans, but also possible causative effects. This approach is used to determine whether ELS disrupts gut and brain homeostasis, and to identify biological pathways that may serve as translatable targets for the prevention or treatment of stress-related psychopathology.

## 2. Materials and Methods

### 2.1. Study Design

Adult male (body weight 400 g) and female (body weight 230–260 g) nulliparous Sprague-Dawley rats were purchased from Charles River Laboratories S.p.A. (Calco, Italy) and allowed a 14-day undisturbed acclimation period at the University of Milan animal facility. Upon arrival, animals were kept in an environment with a 12/12-h light/dark cycle (lights on at 07:00 a.m.), temperature and humidity-controlled room (21 ± 1 °C, 55 ± 5%, respectively) and ad libitum access to food and water. These environmental conditions were maintained throughout the experiment. After acclimatisation, thirty female rats were mated with conspecific males (1 male and 1 female) for two consecutive days. Pregnant rats were housed in pairs until gestational day (GD) 14, after which they were housed individually. Pregnant females were then randomly assigned to stress exposure (PNS, n = 14) or control (CTRL, n = 9) groups. Dams in CTRL group remained undisturbed in their home cages, whereas PNS dams underwent a validated PNS model, involving restraint stress paradigm from GD14 to GD21, as previously described [2,30,32,33]. Briefly, PNS dams were placed into transparent Plexiglas cylinders (20 cm length × 9 cm diameter × 9 cm height) 3 times a day for 45 min (starting at 9 a.m., 12 p.m., and 5 p.m. ± 2 h) under bright light (1500 Lx). After birth, all the offspring were left undisturbed in-home cages with the dam. At postnatal day (PND) 21, offspring were weaned and housed in a group of 2 per cage and were then sacrificed at adulthood (PND84) by decapitation.

Animal handling and experimental procedures were conducted in full accordance with the Italian legislation on animal experimentation (Italian Legislative Decree No. 26 dated 4 March 2014) and adhered to EU recommendations (Legislative Decree No. 26 Implementing Directive 2010/63/EU). The experimental procedure received ethical approval from the Animal Welfare Body (OPBA) of the University of Milan and the Italian Ministry of Health (Approval code: 752/2020-PR; Approval date: 9 April 2020).

### 2.2. Behavioural Analyses

Adult offspring (PND68 to PND78), either exposed or not to PNS, were behaviourally assessed. Here, previously published findings on sociability, assessed via the Social Interaction (SI) Test, are reported [30]. The outcomes obtained enabled the stratification of PNS-exposed animals into resilient (RES) and vulnerable (VULN) phenotypes. Specifically, the test was conducted in an arena (100 cm × 100 cm × 18 cm), which included an empty grid enclosure that allowed sensorial and nose contact between the animals [30,34].

The protocol consisted of two phases, each lasting 3 min: the habituation phase and the test phase. During the habituation phase, the test rat was placed in the arena to freely explore the environment and the empty enclosure. After this phase, the test rat was temporarily returned to its home cage for 1 min. During this interval, an unfamiliar control rat (matched for strain, age, and sex) was placed in the grid enclosure. The test rat was then reintroduced into the arena for the test phase. Social behaviour was evaluated through the SI Ratio, calculated comparing the time spent interacting with the unfamiliar rat to the time spent interacting with an empty cage and is expressed as

SI = ∑ TIZsocial/(∑ TIZsocial + ∑ TIZobject) × 100

where TIZsocial is the time spent in the interaction zone (zone surrounding the grided enclosure) during the test phase, and TIZobject is the time spent in the interaction zone during the habituation phase [35]. To identify the subgroups of RES and VULN animals among the PNS group, a cut-off value was determined as the CTRLs’ mean score minus one standard deviation [36,37]. Animals scoring higher than this cut-off were classified as RES, while those scoring lower were classified as VULN to PNS exposure. Data are presented graphically as means ± standard error of the mean (SEM) in bar plots.

### 2.3. Biological Sample Collection

Brain, small intestine, colon tissues and its luminal content were collected at the sacrifice (PND84); regarding intestinal tissues, 2 cm specimens of intestinal tissue was obtained from sections of the ileum (2–4 cm proximal to the ileocecal junction) and transverse colon, taking care not to include fat residues and washing all segments with phosphate-buffered saline (PBS).

Brain, small intestine, and colon tissues were immediately flash-frozen or fixed in 4% paraformaldehyde and stored at −80 °C, while stool samples were collected into 2 mL tubes and stored at −20 °C until processing.

### 2.4. Immunofluorescence-Based Analyses

Fixed colon samples were placed in plastic moulds containing an embedding medium (Killik OCT Bioptica #05-9801, Bio-Optica Milano S.p.A., Milan, Italy) and then frozen. Samples were sectioned at 10 μm thickness using the HM 525NX cryostat (Thermo Fisher Scientific Inc., Waltham, MA, USA) at −20 °C. For immunofluorescence analyses, anti-ZO1 rabbit antibody, anti-Occludin rabbit antibody (Invitrogen #40-2200, Invitrogen #71-1500; diluted 1:100 and Occludin 1:50 in PBS1x, Invitrogen, Waltham, MA, USA) and Alexa Fluor 488 goat anti-rabbit antiserum (#A322731, Invitrogen, Waltham, MA, USA; diluted 1:200) were used. Images for quantitative analysis were obtained by using confocal laser scanning microscopy, LSM 810 (Carl Zeiss MicroImaging, Oberkochen, Germany) with a 63×/1.4 oil objective. Images were processed with Fiji software (version 1.0), an open-source platform for biological image analysis [38]. Background signals were subtracted by the MosaicSuite plugin (version nov2016), and cleaned images were then analysed by a home-made macro in Fiji. The median of fluorescence intensity was calculated to assess the quantitative presence of antigens in tissues. Data are presented graphically as the median of fluorescence intensity ± SEM in bar plots.

### 2.5. Morphometric Analyses

Intestinal samples were dissected and processed similarly as described for immunofluorescence analyses. Samples were cut in 20 μm thickness. Colon and ileum samples were coloured with Haematoxylin–Eosin (HE) staining (Mayer’s haematoxylin Dako, Carpinteria, CA, USA, #S3309, Eosin Y Dako #CS710) and were imaged using the “Zeiss Axioskop 2 plus” (Carl Zeiss, Oberkochen, Germany) (20× magnification). Measurements of the length and width of crypts and villi height were performed manually using “ISCapture version 3.6.7”. Six intact and well-oriented villi were measured for the ileum, and ten lengths between two complete crypts per rat were considered for colon [39]. The surface area was calculated by using the reported formula [40]: surface area = (villus width × villus length) + (villus width/2 + crypt width/2)^2^/(villus width/2 + crypt width/2)^2^. Data are presented graphically as means ± SEM in bar plots.

### 2.6. Gene Expression Analyses

Total RNA was isolated from the hippocampus and colon tissues using the miRNeasy Mini Kit (Qiagen, Hilden, Germany), according to the manufacturer’s instructions. The RNA concentration and quality were measured at the NanoDrop spectrophotometer (NanoDrop Technologies, Wilmington, DE, USA). Gene expression analyses were performed by using the kit One-Step iTaq Universal Probes (Bio-Rad Laboratories, Hercules, CA, USA) and TaqMan Gene Expression Assays (Applied Biosystem, Thermo Fisher Scientific, Waltham, MA, USA) or Eurofins Genomics probes for the following genes: Cluster of Differentiation 68 (CD68, sequences provided in the Supplementary Table S1), C-X3-C Motif Chemokine Receptor 1 (CX3CR1, sequences provided in the Supplementary Table S1), IL-1β (Assay ID: Rn00580432_m1), IL-6 (Assay ID: Rn01410330_m1), Occludin (Ocln, Assay ID: Rn00580064_m1), TNFα (Assay ID: Rn99999017_m1) and Zonula occludens-1 (ZO-1, Assay ID: Rn07315717_m1) on the CFX384 instrument (Bio-Rad Laboratories, Hercules, CA, USA). Samples were run in triplicate, and each target gene has been normalised to the expression of the housekeeping (HK) genes beta Actin (β-Actin, Assay ID: Rn00580432_m1) or the glyceraldehyde-3-phosphate dehydrogenase (Gapdh, Assay ID: Rn99999916_s1). Data have been analysed using the Pfaffl method to calculate the relative expression ratio of each gene of interest in the different groups [41] and graphically presented as means ± SEM in bar plots.

### 2.7. Gut Microbiome Composition by Bacterial 16S Sequencing

DNA samples were extracted from colon tissues and colon content by using the QIAamp PowerFecal Pro DNA Kit (Qiagen, Hilden, Germany) and by using the QIAamp DNA Stool Mini Kit (Qiagen, Hilden, Germany), respectively, according to the manufacturer’s instructions. DNA was then checked for its concentration and quality by using the NanoDrop spectrophotometer (NanoDrop Technologies, Wilmington, DE, USA) and stored at the temperature indicated in the manufacturer’s instructions until further analysis. The regions V3–V4 of the bacterial ribosomal RNA 16S gene were amplified and purified according to the 16S Metagenomic Sequencing Library Preparation protocol by Illumina (Illumina, San Diego, CA, USA). Amplicon DNA was uniquely dual indexed by using the Nextera XT indices. Indexed DNA was loaded into the MiSeq v3 cartridge (Illumina, Illumina, San Diego, CA, USA). A paired end read of 300 cycles per read was performed with the Metagenomics 16S rRNA application on the MiSeq platform (Illumina, Illumina, San Diego, CA, USA).

Microbial sequences were processed in the Quantitative Insights Into Microbial Ecology 2 (QIIME 2) bioinformatics platform (version 2023.9) [42], run on a Linux workstation (Ubuntu 18.04.5 LTS) equipped with Intel CPU 8 × 3.70 GHz processors and 62.7 GB of RAM. Paired-end sequences were subjected to quality control including denoising, merging, and chimeras removal using the DADA2 plugin with the default parameters [43]. No samples were excluded by the rarefaction step.

The alpha and beta diversity were evaluated using a QIIME 2 pipeline. SILVA reference database (version 138) (https://www.arb-silva.de/, accessed on 1 August 2025), was used to infer the taxonomy of the amplicon sequence variants (ASV) at the phylum and genus level. Finally, the R package MaAsLin2 (Microbiome Multivariable Associations with Linear ModelsLongitudinal, version 1.20.0) with default parameters was used to identify genera associated with PNS. The analyses were assessed separately for each sex. All findings passing an un-adjusted *p* < 0.050 are included in the results.

### 2.8. Statistical Analyses

Statistical analyses have been performed using GraphPad Prism (version 10.0.0) (GraphPad Prism, Boston, MA, USA, www.graphpad.com) and RStudio (version 2023.06.1).

Statistical comparisons for the SI test, morphometric analyses, immunofluorescence, and gene expression were performed using unpaired *t*-tests or one-way ANOVA for normally distributed data, and Mann–Whitney or Kruskal–Wallis tests for non-normally distributed data. The choice of test was based on data distribution and sample size. The z-score for gene expression of pro-inflammatory markers (IL-6, IL1-β, TNFα, CD68 and CX3CR1 in VH and DH) was calculated using the formula: z = (x − μ)/σ, where x is the relative expression ratio of each gene, μ is the mean of the CTRL group, and σ is the standard deviation of the CTRL group. The z-score for each sample was obtained by averaging the individual z-scores of all the genes of interest. To calculate a z-score, qRT-PCR results from at least three genes had to be available for the same animal. For quantitative immunofluorescence analysis, 6 scans for each animal were acquired. Non-parametric Kruskal–Wallis and Mann–Whitney for pairwise group comparison tests were performed in QIIME 2 to determine alpha and beta diversity. Unadjusted associations of the relative abundance of genera, SI score, neuroinflammatory markers, morphometric parameters and TJs (relative gene expression) were assessed by applying Spearman’s rank correlations. Possible outliers were identified and excluded using GraphPad Prism 10.1.0 (ROUT, Q = 1). GraphPad Prism and the Corrplot package (version 0.95) in RStudio were used for graphical visualisation.

## 3. Results

### 3.1. Prenatal Stress Affects the Social Behaviour of Male and Female Adult Offspring

The impact of PNS on behaviour was firstly assessed (Supplementary Figure S1) and no significant differences in the SI ratio between the CTRL and PNS-exposed animals, both males and females, were observed (*p* = 0.268 in males and *p* = 0.126 in females) [30]. However, when stratifying the PNS group for SI ratio, two PNS-exposed subgroups were identified: VULN and RES. A marked reduction in the SI ratio was observed in VULN animals compared to CTRL and RES groups, in both sexes (males: *p* < 0.001 VULN vs. CTRL, *p* < 0.001 VULN vs. RES, and *p* = 0.290 RES vs. CTRL animals; females: *p* < 0.001 VULN vs. CTRL, *p* < 0.001 VULN vs. RES, and *p* = 0.793 RES vs. CTRL animals) [30].

### 3.2. Prenatal Stress Exposure Causes Long-Term Alterations in the Intestinal Architecture

**Figure 1:**
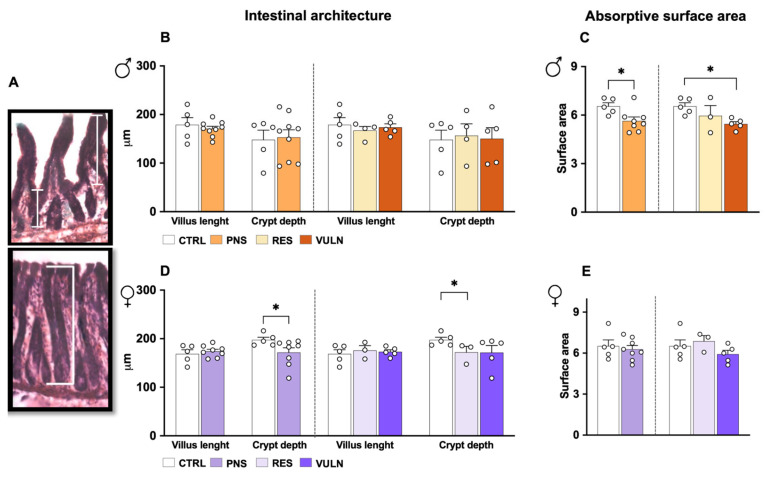
Impact of PNS on gut architecture. (**A**) Representative measurements of ileal villus (**top**) and crypt depth in the colon (**bottom**) from HE-stained sections of adult offspring. Images were captured using a Zeiss Axioskop 2 plus at 20× magnification. Quantification of villus length in the ileum and crypt depth in the colon for male (**B**) and female (**D**) offspring, respectively. (**C**) Measurement of the surface area in the ileum for male and female (**E**) offspring. In each graph, the left bar plots represent the CTRL and PNS comparison. The right bar plots represent the CTRL, RES and VULN comparison. Data are expressed as mean ± SEM. (* *p* < 0.05).

To further investigate potential biological mechanisms underlying the observed sociability outcome, intestinal architecture, permeability, and microbiota composition were examined. Morphological assessment of the gut in male animals exposed to PNS showed no significant changes in ileal architecture, as assessed by villus length measurements (*p* = 0.699), or colonic crypt depth (*p* = 0.999) as compared to CTRL (Figure 1B). Similarly, no significant differences were detected across subgroups in either villus length (*p* = 0.762 VULN vs. CTRL animals, *p* = 0.817 VULN vs. RES animals, and *p* = 0.605 RES vs. CTRL animals) or colonic crypt depth (*p* = 0.880 VULN vs. CTRL animals, *p* = 0.845 VULN vs. RES animals, and *p* = 0.957 RES vs. CTRL animals). However, when assessing the absorptive surface of the ileum through surface area measurements, a significant reduction was observed in PNS animals compared to CTRLs (*p* = 0.030) (Figure 1C). Subgroup analyses indicated that this effect was specifically driven by VULN animals, which showed a significant decrease compared to CTRLs (*p* = 0.015), whereas no significant differences emerged between VULN and RES or between RES and CTRL animals (both *p* = 0.292).

In female animals, ileal morphology was not affected by PNS exposure. Indeed, no significant differences were found in villus length (*p* = 0.724) (Figure 1D) or in the calculated absorptive surface area (*p* = 0.833) (Figure 1E) between PNS-exposed animals and CTRLs. Consistent with these findings, subgroup analyses did not reveal any significant differences in both villus length (*p* = 0.808 VULN vs. CTRL animals, *p* = 0.725 VULN vs. RES animals, and *p* = 0.574 RES vs. CTRL animals) and absorptive surface area (*p* = 0.330 VULN vs. CTRL animals, *p* = 0.101 VULN vs. RES animals, and *p* = 0.426 RES vs. CTRL animals). In contrast, colonic crypt depth was significantly reduced in PNS-exposed animals than CTRLs (*p* = 0.045). Subgroup analysis indicated that this reduction was driven by RES animals, which showed significantly decreased crypt depth compared to CTRLs (*p* = 0.031), while no significant differences were found between VULN and CTRLs (*p* = 0.144) or between VULN and RES animals (*p* = 0.373).

### 3.3. Prenatal Stress Exposure Affects Tight Junctions in Adult Rats

**Figure 2:**
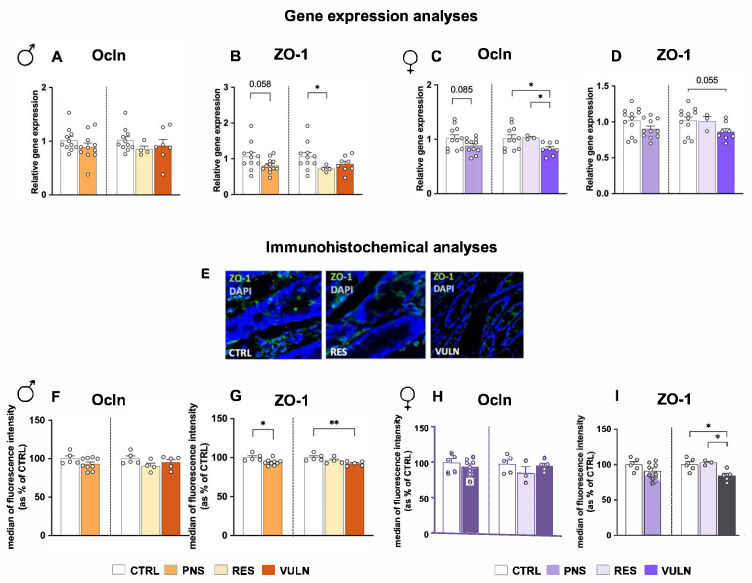
Modulation of TJ-associated molecules in adult animals exposed to PNS and CTRLs. (**A**–**D**) Gene expression of Ocln and ZO-1 assessed by qPCR in male (**A**,**B**), and female (**C**,**D**) animals. (**E**) Representative fluorescence images of colon sections stained with ZO-1 (green) and DAPI (blue) for CTRL, RES, and VULN animals. Images were captured using a confocal laser scanning microscope LSM 810 at 63× magnification. (**F**,**I**) Median of fluorescence intensity of Ocln and ZO-1 in colon sections of males (**F**,**G**), with corresponding data for female animals (**H**,**I**). In each graph, the left bar plots represent the CTRL and PNS comparison. The right bar plots represent the CTRL, RES and VULN comparison. Data are expressed as mean ± SEM in bar plots. (* *p* < 0.05, ** *p* < 0.01).

To characterise intestinal function, epithelium-related parameters were examined by measuring the gene expression of key molecules involved in maintaining epithelial integrity and permeability, specifically TJ markers. In males (Figure 2A,B), no significant differences were observed in Ocln expression between CTRL and PNS-exposed animals (*p* = 0.155), and across subgroups (*p* = 0.374 VULN vs. CTRL animals, *p* = 0.411 VULN vs. RES animals, and *p* = 0.106 RES vs. CTRL animals). In contrast, ZO-1 expression showed a trend toward downregulation in PNS animals compared to CTRLs (*p* = 0.058). Subgroup analyses revealed that this reduction was significantly evident in RES animals (*p* = 0.033), although no significant differences were found between VULN and CTRL (*p* = 0.324) or between VULN and RES animals (*p* = 0.201).

In female offspring (Figure 2C,D), Ocln expression showed a trend toward reduction in the PNS group compared to CTRLs (*p* = 0.085). This effect was driven by VULN animals, which showed significantly lower relative gene expression compared to both CTRL (*p* = 0.026) and RES groups (*p* = 0.027), whereas no significant differences were observed between RES and CTRL groups (*p* = 0.484). For ZO-1, no significant differences were observed between PNS and CTRL groups (*p* = 0.105). However, subgroup analyses revealed a trend toward reduced expression in VULN animals compared to CTRL (*p* = 0.055), while differences between VULN and RES (*p* = 0.232) and between RES and CTRL (*p* = 0.917) were not significant.

To gain a comprehensive understanding of the impact on epithelial barrier functions, gene expression results were integrated with immunohistochemical analyses. In PNS-exposed male animals (Figure 2F,G), no significant differences were observed in Ocln expression between PNS and CTRL groups (*p* = 0.178), and subgroup analyses also revealed no significant effects among VULN, RES, and CTRL animals (*p* = 0.472 VULN vs. CTRL animals, *p* = 0.273 VULN vs. RES animals, and *p* = 0.076 RES vs. CTRL animals). Conversely, ZO-1 expression was significantly reduced in PNS animals compared to CTRLs (*p* = 0.012), with this effect being particularly pronounced in the VULN subgroup (*p* = 0.006 VULN vs. CTRL), while no significant differences were found between VULN and RES (*p* = 0.214) or RES and CTRL (*p* = 0.172) animals.

Comparable results were observed in females (Figure 2H,I). No significant differences in Ocln expression were observed between PNS and CTRL groups (*p* = 0.524), or among the subgroups (*p* = 0.871 for VULN vs. CTRL animals, *p* = 0.251 for VULN vs. RES animals, and *p* = 0.197 for RES vs. CTRL animals). In contrast, while ZO-1 expression was not significantly reduced in the overall PNS group compared to CTRL (*p* = 0.182), subgroup analysis revealed a significant downregulation in VULN animals compared to both CTRL (*p* = 0.025) and RES (*p* = 0.024) animals. No significant difference was found between RES and CTRL animals (*p* = 0.742).

### 3.4. Prenatal Stress Exposure Drives Neuroinflammatory Responses in Dorsal and Ventral Hippocampus

Following the assessment of intestinal architecture and building on previous evidence on PNS-induced neuroinflammation, this response was further investigated in the context of gut–brain axis communication. As previously described [30], PNS exposure led to an increase in inflammatory markers (IL-1β, TNFα) and of microglial activation markers (CD68 and CX3CR1) in the VH of male VULN animals (Supplementary Figure S2). This inflammatory profile was further supported by z-score analysis, which confirmed a general upregulation of inflammation-related genes in VULN animals compared to both CTRL and RES groups. The same markers were also measured in the VH of female offspring [30]; however, no significant differences were observed (Supplementary Figure S3).

In addition to the VH, the dorsal hippocampus (DH) was also assessed here. In male offspring (Supplementary Figure S4), IL-6 levels did not significantly differ between CTRL and PNS groups (*p* = 0.922), and across subgroups (*p* = 0.506 VULN vs. CTRL animals, *p* = 0.272 VULN vs. RES animals, and *p* = 0.806 RES vs. CTRL animals). In contrast, IL-1β showed no significant difference between CTRL and PNS groups (*p* = 0.918) but was significantly upregulated in VULN animals compared to RES (*p* = 0.024), with no significant differences between VULN and CTRL (*p* = 0.229) or RES and CTRL (*p* = 0.817) animals. A comparable trend was observed for TNFα, where the overall comparison between CTRL and PNS was not significant (*p* = 0.586), however VULN animals showed significantly higher levels than both CTRL and RES animals (*p* = 0.007 VULN vs. CTRL animals, *p* < 0.001 VULN vs. RES animals, and *p* = 0.246 RES vs. CTRL animals). These inflammatory changes, as assessed by cytokine levels, are further supported by increased expression of microglia-related genes. Specifically, for CD68, no significant difference was found between CTRL and PNS groups (*p* = 0.905). However, VULN animals showed significantly higher expression compared to RES (*p* = 0.050), while no significant differences were observed between VULN and CTRL (*p* = 0.356) or RES and CTRL (*p* = 0.385). Similarly, for CX3CR1, the relative gene expression did not significantly differ between CTRL and PNS (*p* = 0.661) but was significantly upregulated in VULN animals compared to RES (*p* = 0.012). No significant differences were found between VULN and CTRL (*p* = 0.066) or RES and CTRL (*p* = 0.525) animals. Z-score analysis confirmed this inflammatory profile, showing an overall upregulation in VULN animals compared to both CTRL and RES groups (*p* < 0.001 VULN vs. CTRL animals, *p* < 0.001 VULN vs. RES animals, and *p* = 0.239 RES vs. CTRL animals).

In female animals, consistent with previous findings in the VH [30], analysis of the DH revealed no statistically significant differences in the expression of most inflammatory markers (all *p* > 0.05) (Supplementary Figure S5). An exception was observed for TNFα, which, although not significantly different between CTRL and PNS groups (*p* = 0.145), was significantly upregulated in RES animals compared to both CTRL (*p* = 0.003) and VULN animals (*p* = 0.001). No significant difference was found between VULN and CTRL groups (*p* = 0.581).

### 3.5. Prenatal Stress Causes a Taxonomic Shift in the Gut Microbiota Composition in the Crypt and Luminal Content of Adult Male Rats

Considering the close relationship between the luminal content of the colon with the epithelial layer, 16S rRNA analyses were performed on the crypt content and luminal samples.

#### 3.5.1. Crypt Content Analyses

**Figure 3:**
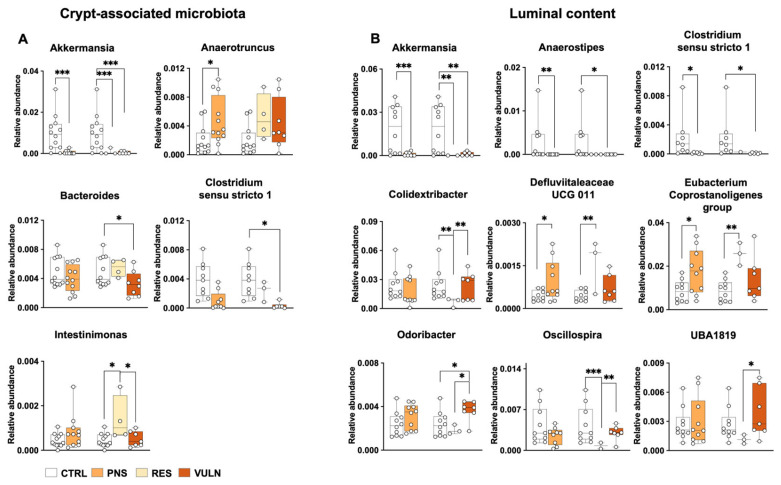
Impact of PNS on the taxonomic composition of crypt-associated microbiota and luminal content in male adult offspring at the genus level. Taxonomic profiles of crypt-associated microbiota (**A**) and luminal content (**B**) identified using MaAsLin2, illustrating microbial differences among the groups. In each graph, the left bar plots represent the CTRL and PNS comparison. The right bar plots represent the CTRL, RES and VULN comparison. Data are presented as relative abundance, with only taxa having a relative abundance >0.01 shown. (* *p* < 0.05, ** *p* < 0.01, *** *p* < 0.001).

Alpha and beta diversity in crypt microbiota indicated no significant differences in male animals exposed or not to PNS (Supplementary Figure S6). However, examining taxonomic differences among groups (Supplementary Table S2) revealed that, at the phylum level, *Verrucomicrobiota* was markedly reduced in PNS animals compared to CTRL (*p* = 0.001), and this reduction was observed both in the RES and VULN subgroups (*p* ≤ 0.002) compared to CTRLs. Conversely, *Patescibacteria*, which were significantly more abundant in PNS compared to CTRL animals (*p* = 0.041), were also significantly enriched in VULN animals (*p* = 0.018) compared to CTRLs. At genus level (Figure 3A; Supplementary Table S3), CTRLs exhibited higher relative abundances of different taxa, including *Akkermansia*, *Anaerostipes*, *Cutibacterium*, and *Lachnospiraceae UCG 008* (all *p* ≤ 0.049) compared to the PNS group. In contrast, *Anaerofustis*, *Anaerotruncus*, *Anaerovorax*, *Auricoccus-Abyssicoccus*, *Candidatus Saccharimonas*, and *Corynebacterium* were more abundant in PNS-exposed animals versus CTRLs (*p* ≤ 0.044).

To further investigate subgroup differences, RES and VULN animals were compared. *Intestinimonas* was significantly more abundant in the crypt-associated microbiota of RES animals compared to both CTRL and VULN groups (*p* ≤ 0.027). In contrast, *Akkermansia* was significantly less abundant in both RES and VULN animals (*p* ≤ 0.001). Furthermore, VULN animals showed reduced levels of *Bacteroides* and *Clostridium sensu stricto 1* compared to CTRLs (*p* ≤ 0.050). The complete list of genera showing significant differences is provided in Supplementary Tables S2 and S3.

#### 3.5.2. Luminal Content Analyses

Consistent with findings in the crypt compartment, alpha and beta diversity analyses of the luminal microbiota revealed no significant differences between PNS-exposed and CTRL males, including within stratified subgroups (Supplementary Figure S7). Comparable results were also observed at the phylum level (Supplementary Table S2), including a decrease in the relative abundance of *Verrucomicrobia* in PNS-exposed animals (*p* = 0.001) and an increase in *Patescibacteria* (*p* = 0.002) compared to CTRLs. Subgroup analyses further supported the alterations observed in the crypt microbiota, showing lower abundance of *Verrucomicrobia* in both RES and VULN animals compared to CTRL (*p* ≤ 0.006), while *Patescibacteria* were more abundant in VULN animals than in CTRLs (*p* = 0.001). Examination of microbiota composition at the genus level revealed significant findings consistent with those observed in the crypt microbiota (Figure 3B; Supplementary Table S3). Specifically, among taxa showing significant differences in relative abundance, PNS exposure was associated with a marked reduction in *Akkermansia*, *Anaerostipes*, and *Clostridium sensu stricto 1* (*p* ≤ 0.011) compared to CTRLs. Conversely, *Defluviitaleaceae UCG011* and *Eubacterium Coprostanoligenes group* were found with a higher relative abundance in the PNS group (*p* ≤ 0.028).

Stratifying according to the behavioural phenotype, it was found that RES animals exhibited a significant increase in the relative abundance of *Defluviitaleaceae UCG011* and *Eubacterium Coprostanoligenes group* (*p* ≤ 0.007), alongside a decrease in other taxa, including *Akkermansia*, *Colidextribacter*, and *Oscillospira* (*p* ≤ 0.006) compared to CTRLs. In contrast, VULN animals displayed reduced levels of *Akkermansia*, *Anaerostipes*, and *Clostridium Sensu Stricto 1* (*p* ≤ 0.025), while *Monoglobus* and other genera, including *Odoribacter*, were more abundant (*p* ≤ 0.015). Furthermore, specific bacterial taxa, namely *Colidextribacter*, *Odoribacter*, *Oscillospira*, and *UBA1819*, were significantly more abundant in VULN than in RES animals (*p* ≤ 0.026). The complete list of genera showing significant differences is provided in the Supplementary Tables S2 and S3.

### 3.6. Prenatal Stress Modulates Gut Microbiota in the Crypt and Luminal Content of Adult Female Rats

#### 3.6.1. Crypt Content Analyses

**Figure 4:**
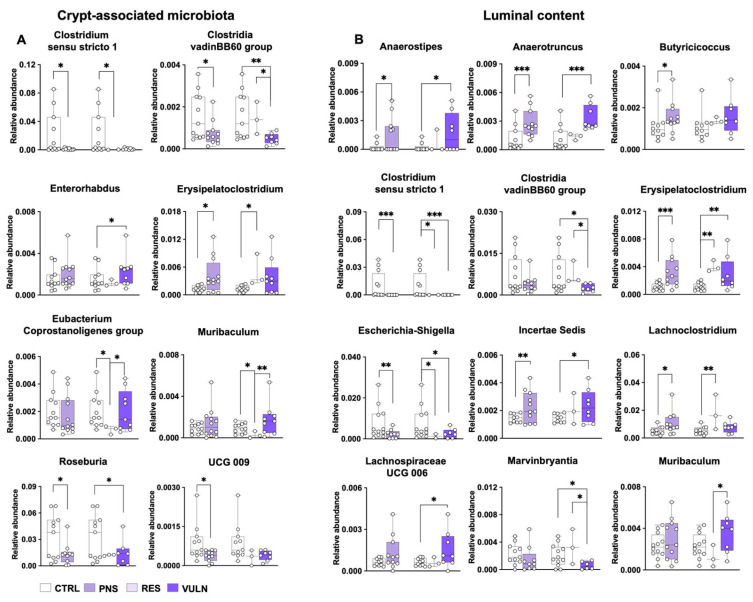
Impact of PNS on the taxonomic composition of crypt-associated microbiota and luminal content in female adult offspring at the genus level. Taxonomic profiles of crypt-associated microbiota (**A**) and luminal content (**B**) identified using MaAsLin2, illustrating microbial differences among the groups. In each graph, the left bar plots represent the CTRL and PNS comparison. The right bar plots represent the CTRL, RES and VULN comparison. Data are presented as relative abundance, with only taxa having a relative abundance >0.01 shown. (* *p* < 0.05, ** *p* < 0.01, *** *p* < 0.001).

Similar analyses conducted in males were also performed in females. Consistent with previous analyses, examination of alpha and beta diversity revealed that these metrics (Supplementary Figure S6) remained unchanged, and no significant alterations were detected at the phylum level in crypt microbiota. However, examination of genus-level changes in the crypt microbiota (Figure 4A; Supplementary Table S4) revealed that CTRLs exhibited higher relative abundances of several taxa, including *Clostridium Sensu Stricto 1*, *Clostridia vadinBB60 group*, *Roseburia*, and *UCG-009* compared to the PNS group (*p* ≤ 0.050). In contrast, the PNS group was characterised by a higher relative abundance of different taxa, among which *Erysipelatoclostridium* (*p* = 0.015). When stratified for behavioural outcomes, RES animals exhibited significantly lower relative abundances of different taxa, including *Clostridium Sensu Stricto 1*, *Eubacterium Coprostanoligenes group*, and *Muribaculum* compared to CTRLs (*p* ≤ 0.047). Similarly, other taxa, including *Erysipelatoclostridium*, have been reported to be more abundant in RES animals (*p* = 0.021) in comparison with CTRL animals.

In addition, comparative analyses between VULN and CTRL animals revealed further significant differences in taxon abundance. Specifically, the *Clostridia vadinBB60 group* and *Roseburia* were found to be more abundant in CTRLs (*p* ≤ 0.019). In contrast, *Enterorhabdus* was more abundant in VULN animals (*p* = 0.048). Further analysis between RES and VULN revealed distinct taxonomic differences associated with each phenotype. For example, *Clostridia vadinBB60 group* was significantly more abundant in RES animals (*p* = 0.020) while *Eubacterium Coprostanoligenes group* and *Muribaculum* were more abundant in VULN animals (*p* ≤ 0.047). The complete list of genera showing significant differences is provided in the Supplementary Tables S2 and S4.

#### 3.6.2. Luminal Content Analyses

As in male offspring, colonic luminal content was also analysed in females. While no significant differences in alpha and beta diversity were detected in colonic crypts, luminal microbiota composition showed group-specific alterations (Supplementary Figure S7). In particular, beta diversity analyses revealed a significant increase in PNS-exposed animals compared to CTRLs, as shown by higher unweighted UniFrac and Jaccard distances (*p* ≤ 0.029). Subgroup analyses revealed that the increased beta-diversity distance, as captured by the Jaccard metric, was primarily driven by the VULN subgroup (*p* = 0.019).

Taxonomic comparisons at the phylum level (Supplementary Table S2) showed that *Desulfobacterota* was significantly more abundant in the PNS group compared to CTRLs (*p* = 0.020). Subgroup analysis revealed that this difference was specifically driven by VULN animals, who exhibited significantly higher levels of this phylum compared to CTRLs (*p* = 0.014). In contrast, *Patescibacteria* was less abundant in PNS-exposed animals (*p* = 0.015), with the lowest levels observed in the VULN subgroup compared to CTRL (*p* = 0.010).

Examination of the luminal content at genus level (Figure 4B; Supplementary Table S4) revealed significant differences in the relative abundances of *Anaerostipes*, *Anaerotruncus*, *Butyricicoccus*, *Erysipelatoclostridium*, *Incertae Sedis*, and *Lachnoclostridium*, all of which were more abundant in PNS animals compared to CTRLs (*p* ≤ 0.034). Conversely, CTRL animals exhibited higher relative abundances of other taxa, including *Clostridium Sensu Stricto 1* and *Escherichia-Shigella*, compared to the PNS group (*p* ≤ 0.008). Similarly to what was observed in the crypt-associated microbiota, specific differences in the luminal content were also particularly pronounced in animals with the RES or VULN phenotype. For instance, RES animals displayed an increased relative abundance of *Erysipelatoclostridium* and *Lachnoclostridium* (*p* ≤ 0.009), and decreased abundance of *Clostridium Sensu Stricto 1* and *Escherichia-Shigella* in the comparison with CTRLs (*p* ≤ 0.012). The VULN phenotype, on the other hand, was characterised by an increased abundance of different taxa, including *Anaerostipes*, *Anaerotruncus*, *Defluviitaleaceae UCG011*, *Desulfovibrio*, *Erysipelatoclostridium*, *Incertae Sedis*, and *Lachnospiraceae UCG 006* (*p* ≤ 0.034) as well as lower abundances of *Clostridium Sensu Stricto 1* and *Escherichia-Shigella* (*p* ≤ 0.035) compared to CTRLs. The comparison between the subgroup of RES and VULN highlights a higher relative abundance of *Clostridium Methylpetosum group* and *Muribaculum* in the VULN animals compared to RES (*p* ≤ 0.036). Additionally, VULN animals exhibited a decrease in *Clostridia vadinBB60 group* and *Marvinbryantia*, both than CTRL and RES animals (all *p* ≤ 0.034). The complete list of genera showing significant differences is provided in the Supplementary Tables S2 and S4.

### 3.7. Spearman Correlation Analysis Connects Stress-Induced Microbiota Shifts Were Associated with Behavioural Outcomes, Neuroinflammation, and Intestinal Parameters in Adult Offspring

**Figure 5:**
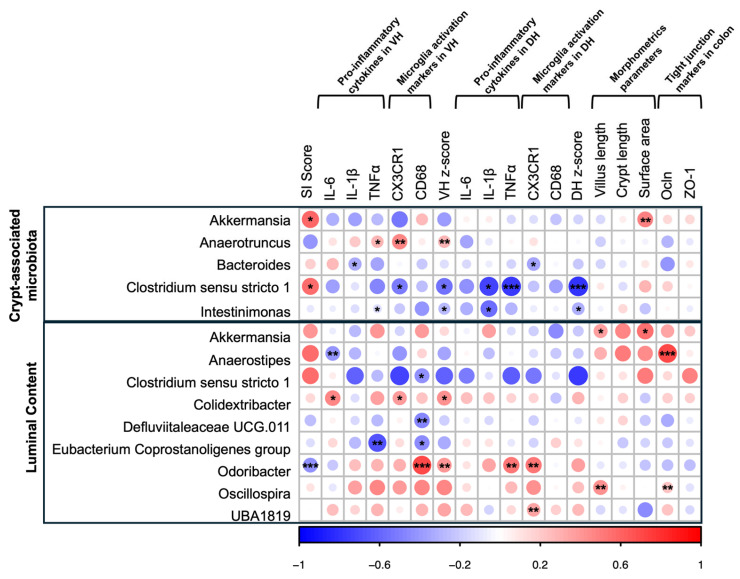
Spearman’s rank correlation matrix of differentially abundant genera with SI score, neuroinflammatory and morphometric parameters, and TJ markers in male rats. The matrix shows Spearman rank correlations, with the colour gradient indicating the strength and direction of the relationship: +1 represents a perfect positive correlation and −1 a perfect negative correlation. Due to the sample size, a threshold of *p* < 0.10 was considered for significance in this analysis (* *p* < 0.10, ** *p* < 0.05, *** *p* < 0.01).

Considering the data on the gut and its association with behavioural outcomes, further investigation was conducted to assess whether these gut alterations are linked to changes in brain inflammatory status, particularly in the hippocampus. In male animals (Figure 5; Supplementary Table S5), taxa identified as differentially abundant in the colonic crypt microbiota showed that “protective” genera such as *Akkermansia* positively correlated with behavioural scores and the surface area (ρ ≤ 0.599, *p* ≤ 0.061). Likewise, short-chain fatty acids (SCFAs)-producing genera, including *Bacteroides*, *Clostridium sensu stricto 1*, and *Intestinimonas*, were negatively associated with pro-inflammatory cytokines, microglial markers, or the corresponding z-scores in both the VH and DH regions (all *p* ≤ 0.096). In contrast, *Anaerotruncus* showed a positive correlation with inflammatory markers in the VH (ρ ≤ 0.465, *p* ≤ 0.087), indicating a distinct association profile compared to SCFA-producing genera.

Correlation analysis of taxa differentially abundant in the luminal microbiota also revealed different significant correlations. Among these, *Anaerostipes* was negatively correlated with IL-6 levels in the VH (ρ = −0.419, *p* = 0.036) and positively associated with Ocln gene expression in the colon (ρ = 0.719, *p* = 0.001). Additionally, *Odoribacter* was negatively associated with SI scores (ρ = −0.425, *p* = 0.007) and positively correlated with inflammatory markers in both VH and DH (ρ ≤ 0.718, *p* ≤ 0.046).

**Figure 6:**
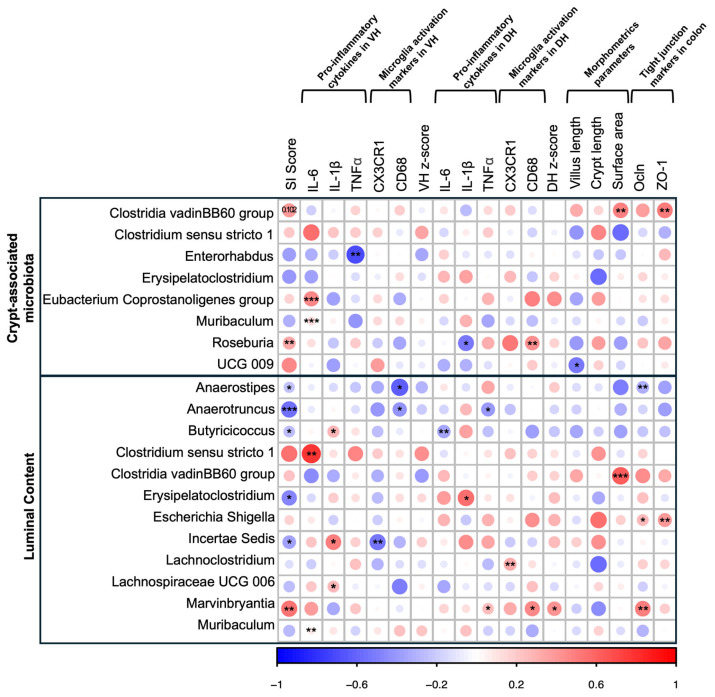
Spearman’s rank correlation matrix of differentially abundant genera with SI score, neuroinflammatory and morphometric parameters, and TJ markers in female rats. The matrix shows Spearman rank correlations, with the colour gradient indicating the strength and direction of the relationship: +1 represents a perfect positive correlation and −1 a perfect negative correlation. Due to the sample size, Due to the sample size, a threshold of *p* < 0.10 was considered for significance in this analysis (* *p* < 0.10, ** *p* < 0.05, *** *p* < 0.01).

The same analyses were performed in female offspring (Figure 6; Supplementary Table S6), revealing significant correlations between group-differentiating taxa in the crypt-associated microbiota and behavioural, neuroinflammatory, and morphological parameters. For example, “protective” bacteria such as *Clostridia vadinBB60* group showed a positive trend with the SI ratio (ρ = 0.394, *p* = 0.102), as well as significant positive associations with the surface area and ZO-1 (ρ ≤ 0.510, *p* ≤ 0.050). Similarly, *Roseburia*, an SCFA-producing genus, was positively correlated with SI scores (ρ = 0.332, *p* = 0.034) and negatively associated with IL-1β measured in the DH (ρ = −0.529, *p* = 0.071). Analyses of differentially abundant taxa in the intestinal lumen also revealed different significant associations. *Anaerostipes*, for instance, was negatively correlated with SI scores, CD68 measured in the VH, and Ocln expression in the colon (ρ ≤ −0.625, *p* ≤ 0.067). On the other hand, some inflammatory markers evaluated in the VH and DH were positively correlated with different taxa, including *Clostridium sensu stricto 1*, *Erysipelatoclostridium*, *Incerta sedis*, *Lachnoclostridium*, *Lachnospiraceae UCG-006*, and *Marvinbryantia* (all ρ ≤ 0.785, *p* ≤ 0.099). The full results, including corresponding *p*-values, are presented in Figure 5 and Figure 6 and in the Supplementary Tables S5 and S6.

## 4. Discussion

This study examines the long-term effects of PNS, with a focus on intestinal barrier integrity and gut microbiota in adult male and female offspring. The results showed that, in addition to inducing behavioural alterations and neuroinflammation as previously observed [30], PNS also produced significant changes in the intestinal architecture, gut permeability markers, and the microbial composition.

Indeed, as previously shown, both male and female rats exposed to PNS exhibited impairments in social behaviour, with a more pronounced effect in VULN animals [30]. Related to this, studies examining the long-term effects of PNS on the brain and behaviour have identified alterations in key brain regions involved in regulating emotions, memory, and learning, such as the amygdala and hippocampus [44,45]. Notably, also a clinical study on young adults born from mothers who experienced stressful life events during the first half of pregnancy showed reduced amygdala volume and a higher susceptibility to mood disorders [46]. In this context, neuroinflammation has emerged as a key mechanism underlying various psychiatric disorders, as it disrupts normal brain function and contributes to behavioural alterations [47,48]. Consistent with this, previous observations of neuroinflammation in the VH [30] and DH of male VULN animals supports this clinical evidence. Interestingly, female PNS-exposed offspring did not display the neuroinflammatory profile observed in male VULN animals, suggesting a sex-specific mechanism associated with the long-term effects of PNS vulnerability and resilience in the two sexes. This protective mechanism may be mediated by gonadal hormones, particularly estrogens, which are known to modulate microglial activation, reduce the production of pro-inflammatory cytokines, and regulate neuroimmune signalling pathways [49,50]. These findings highlight a critical interaction between sex and PNS, pointing to distinct biological trajectories in males and females, and underscore the importance of further research to elucidate the mechanisms underlying the stress-related neuroimmune outcomes.

To deepen understanding of these mechanisms, intestinal morphology was examined [51], as disruptions in gut structure may contribute to systemic inflammation and altered neuroimmune signalling [52]. The results revealed a long-term impact of PNS on gut architecture, evidenced by decreased crypt depth in female animals. Although the long-term effects of PNS on the intestinal barrier are still limited, previous studies have indicated that PNS impairs the gut microarchitecture, the cell turnover and the mucosal barrier function in 3-week-old offspring [25]. These changes suggest a reduced intestinal physiological efficiency, potentially linking gastrointestinal abnormalities with negative behavioural outcomes. Such alterations frequently co-occur in psychiatric conditions, where gut dysfunctions are often observed alongside with symptoms of anxiety and depression [53,54]. Moreover, shortened crypts may impair the gut’s ability to defend against pathogens and maintain immune homeostasis [55,56]. Since preserving the integrity of the intestinal barrier is essential for a proper gut–brain axis functioning [57], increased focus has been placed on gut permeability changes and their association with psychiatric conditions [52].

Investigation of the epithelial architecture revealed a reduction in the surface area of the ileal mucosa in adult male animals exposed to PNS. Importantly, this reduction was evident only in animals displaying the VULN phenotype. The surface area of the small intestine is largely determined by the height and density of villi, which play a crucial role in increasing absorptive capacity and supporting nutrient and electrolyte uptake [58], and alterations in this structure may, therefore, compromise intestinal function. Gastrointestinal structure and function can be disrupted by ELS, leading to compromised epithelial barrier integrity and increased intestinal permeability [25]. Notably, male PNS offspring exhibited a reduced ileal mucosal surface, whereas female PNS offspring showed a shortened crypt depth in the colon. This sex-dependent modulation of the gut barrier in response to PNS could, for example, reflect estrogen-mediated structural adaptations in females [59]. Maintaining an adequate absorptive surface is essential for intestinal function, both to ensure nutrient uptake and to prevent the translocation of potentially pathogenic bacteria [60,61]. A central role in limiting paracellular translocation and preserving intestinal homeostasis is played by TJs [62], particularly ZO-1.

In this context, a significant downregulation of ZO-1 in both male and female offspring exposed to PNS was observed, as assessed through immunofluorescence. By stratifying animals based on their behavioural phenotype, it was further demonstrated that this reduction was specific to VULN animals, suggesting a mechanistic link between stress-induced intestinal barrier dysfunction and behavioural vulnerability. Similarly, a comparable downregulation was observed for Ocln in female offspring, as indicated by gene expression analyses. These results are in line with the effects produced using a psychological stress model in adult animals that revealed decreased levels of intestinal TJ proteins, such as claudin-5, Ocln, α-actin, and ZO-1, in stressed animals as compared to CTRLs [63]. Another study, using an animal model of depression based on chronic social and variable stress, demonstrated that the expression of TJ genes in the jejunum is modulated by both the type and duration of stress exposure [64]. This highlights how different stress paradigms can exert distinct effects on gut epithelial integrity, with chronic or unpredictable stressors potentially leading to prolonged molecular changes in barrier function. Such findings align with the growing body of evidence suggesting that psychological stress can disrupt TJ integrity, thereby increasing intestinal permeability, which in turn may not only compromise gut function, but also promote a pro-inflammatory state, contributing to a cascade of biological alterations. Interestingly, recent studies have emphasised the pivotal role of TJ proteins in the interplay between gut dysbiosis and mental health disorders [65,66], reinforcing the idea that the gut barrier acts as a key interface in the gut–brain axis. Building on this, the observed downregulation of TJ components in stress-vulnerable animals may reflect a persistent impairment of intestinal barrier integrity. This disruption could ultimately compromise gut–brain communication and potentially contribute to long-term psychiatric vulnerability.

This study also examined potential differences in microbiota composition associated with the PNS exposure by studying the crypt-associated microbiota and luminal content of the colon. Distinct abundance levels of several bacterial taxa were observed across the groups. Although CTRL animals showed an increased relative abundance of specific bacteria such as *Akkermansia*, *Bacteroides*, and *Anaerostipes* in males, and *Clostridia vadinBB60 group* and *Roseburia* in females, the specific pathways through which these bacteria influence the intestinal barrier and neuroinflammation likely involve their metabolic products, particularly SCFAs. The main SCFAs are acetate, propionate, and butyrate, which are essential metabolites for maintaining host health [67] and play a crucial role in preserving intestinal barrier integrity, supporting mucin production, modulating local immune responses, including regulatory T cell differentiation and suppression of pro-inflammatory cytokines [68,69]. Notably, lower levels of SCFAs, particularly butyric acid, have been associated with the disruption of TJs, increased gut permeability and heightened intestinal inflammation [70]. Consistently, SCFAs have been reported to be higher in CTRL animals compared to those exhibiting abnormal behaviours [68,71] and have also been shown to decrease in response to ELS [72]. Contrarywise, SCFA supplementation alleviates symptoms of anhedonia and reduces heightened stress responsiveness [73]. The positive correlations observed between specific SCFA-producing bacteria, social behaviour, and TJs, in this study, likely support the beneficial effects of these metabolites on the epithelial barrier and behaviour, particularly considering that these aspects remained undamaged in CTRL animals. Additionally, supporting the possible role of these bacteria in the development of specific behavioural phenotypes, an increase in the relative abundance of SCFA-producing bacteria in RES animals was observed, such as the genus *Intestinimonas* in the crypt of male animals and *Clostridia vadinBB60 group*, in the crypt and luminal content of females, when compared to VULN phenotype animals. Although the beneficial effects of SCFAs on behaviour and brain function have already been demonstrated [74], only a few studies have specifically examined the microbiota changes in association with stress resilience and vulnerability, especially in the context of PNS. Indeed, Han and colleagues reported that SCFA-producing bacteria, such as the genus *Roseburia*, are more abundant in RES animals exposed to a chronic unpredictable mild stress [75]. Moreover, the finding that SCFA production can reverse stress-induced aberrant behaviour, likely through their anti-inflammatory effects, suggests that the underlying mechanism may involve modulation of the inflammatory system, a well-established mediator in behavioural and brain alterations. This insight underscores the importance of further distinguishing between RES and VULN animals, as well as understanding how inflammation contributes to these distinct phenotypes.

In this study, although male VULN animals exhibited some protective bacteria such as *Oscillospira* [76], a higher abundance of pro-inflammatory bacteria was also observed, including the genus *Anaerotruncus* among PNS-exposed males and female animals, and notably, in VULN females. In addition to the animal models proposed by Ding and colleagues [77], human studies have also reported a higher abundance of pro-inflammatory genera in psychiatric patients compared to healthy controls [78,79]. These findings suggest that inflammatory processes derived from the gut microbiota can be involved in neuroinflammation, a condition observed also in these PNS-exposed animals. The genus *Anaerotruncus*, besides having pro-inflammatory effects, compromises the integrity of the epithelial barrier [80]. These alterations are known to promote low-grade systemic inflammation through the translocation of inflammatory molecules and microbial components, including bacterial products, into the systemic circulation [81]. This process can further lead to the crossing of the blood–brain barrier, initiating a cascade of events that impact the central nervous system (CNS) [82]. Therefore, in addition to assessing dysbiosis, studying the specific composition of the gut microbiota and its relation to specific central biomarkers could help elucidate how disruptions in the bacterial community may compromise brain functions.

To explore this interconnection, the associations between differentially abundant bacterial taxa and neuroinflammatory markers measured in both the VH and DH were further examined. Notably, in male animals, beneficial bacteria such as *Bacteroides* showed negative correlations with microglial activation and inflammatory markers in the VH and DH, whereas pro-inflammatory taxa like *Anaerotruncus* were positively associated with these markers. These findings are in line with a previous study [33] where a negative correlation was found between the health-promoting bacteria such as *Christensenellaceae R7 group* [83], microglial activation and inflammatory markers such as CR3CL1, CD68 and IL-6 evaluated in the DH of animals exposed to social isolation during adolescence. Microglia are essential for maintaining neural homeostasis, regulating synaptic plasticity, neurogenesis, and responding to brain injury [84], and the DH is particularly sensitive to immune dysregulation and stress-coping strategies [85,86]. Alterations in its microglial profile may thus reflect, or even mediate, long-term behavioural outcomes linked to stress. Given these critical functions and the recognition that peripheral inflammatory mediators can trigger microglial activation, affect brain function, and ultimately impact behaviour [87], it is important to further explore these mechanisms and the gut microbiota to better understand how to modulate negative responses to stress early in life.

These findings highlight the gut microbiota and intestinal barrier integrity as key mediators of the long-term impact of ELS on brain function and behaviour. Moreover, they contribute to a growing body of evidence suggesting that early disruptions in gut–brain axis pathways may shape later vulnerability or resilience to stress-related disorders. Animal models, indeed, allow the analyses of specific variables and features of the brain and the gut that are not easily accessible in human populations. Moreover, animal studies enable not only to identify associations, as in humans, but also possible causative effects. Through this approach, this study aims to determine whether ELS disrupts gut and brain homeostasis and to identify biological pathways that may serve as translatable targets for the prevention or treatment of stress-related psychopathology.

In this context, translational studies are needed to determine whether similar alterations identified in the PNS model also occur in humans, and to what extent these preclinical findings can inform the development of microbiota-targeted interventions in at-risk populations. Characterizing these pathways opens the way to microbiota-targeted interventions, such as the use of specific probiotics or prebiotics designed to promote beneficial bacterial taxa, dietary strategies to support microbial diversity, and complementary approaches aimed at restoring microbial balance, enhancing gut barrier integrity, and modulating both systemic and neuroinflammatory responses. Such interventions could be further refined based on individual microbiome profiles, ultimately enabling personalized approaches to prevent or mitigate stress-related psychiatric conditions. Importantly, future studies using causal approaches, such as fecal microbiota transplantation or targeted probiotic interventions, will be essential to validate these strategies and establish direct links between microbial modulation, intestinal barrier function, and neuroimmune outcomes. Identifying microbial or intestinal biomarkers associated with vulnerability or resilience may also pave the way for early screening tools and personalised treatments in stress-related psychiatric conditions.

This study has some limitations. The first one is represented by coprophagists as animals shared the same cage, and therefore, it is possible that they ingested faecal material from other animals, thus introducing a variable that may have affected the microbiota composition and subsequently the results. Secondly, the use of 16S rRNA sequencing restricts the analysis to genera, which encompasses a variety of species and strains of bacteria that could play distinct metabolic and regulatory roles. This approach also suffers from reduced taxonomic resolution and cannot directly assess functional activity, unlike metagenomic approaches. In addition, we adopted a more lenient threshold (*p* < 0.1) for correlation analyses, which may increase the likelihood of false-positive associations. Lastly, while our study did not directly assess inflammatory status, further research is needed to establish its relationship with the observed changes. To overcome these limitations and obtain more comprehensive insights, future research will need to include a larger sample size, which may increase the statistical power, particularly in subgroup analyses and integrate these results with longitudinal observation and the immune system.

## 5. Conclusions

Stress during critical windows of development is well-known for its harmful effects on the developing CNS, making it essential to investigate its long-term consequences. Previous and current findings demonstrate that PNS exerts long-term effects on brain function and behaviour, significantly affecting gut microbiota composition and gut architecture, which may lead to altered intestinal permeability. Importantly, these effects differ between male and female offspring, suggesting sex-specific responses to PNS. Furthermore, considering the differences observed between RES and VULN rats exposed to PNS, these data reflect the notion that not all the individuals exposed to ELS will develop negative mental outcomes and that changes in the gut microbiota composition and gut architecture may be involved in the onset of resilience or vulnerability.

Overall, this study contributes to the growing body of evidence linking ELS to behavioural disorders, emphasising the critical role of the gut, its architecture and its microbial community, and its impact on central inflammation.

## Data Availability

All data generated during and/or analyzed are available from the corresponding author.

## Supplementary Materials

The following supporting information can be downloaded at https://www.mdpi.com/article/10.3390/nu17172812/s1, Figure S1: Prenatal stress affects the sociability; Figure S2: Gene expression analysis of inflammation-related and microglia activation markers in the ventral hippocampus of male offspring; Figure S3: Gene expression analysis of inflammation-related and microglia activation markers in the ventral hippocampus of female offspring; Figure S4: Gene expression analysis of inflammation-related and microglia activation markers in the dorsal hippocampus of male offspring; Figure S5: Gene expression analysis of microglia activation and inflammation-related markers in the dorsal hippocampus of female offspring; Figure S6: Analysis of alpha and beta diversity of the crypt microbiota in male and female offspring; Figure S7: Analysis of alpha and beta diversity of the crypt microbiota in female and female offspring; Table S1: Primers and probes sequences; Table S2: Significant phylum-level differences in crypt and lumen by group and sex; Table S3: Significant genus-level microbial differences in crypt and lumen of male offspring; Table S4: Significant genus-level microbial differences in crypt and lumen of female offspring; Table S5: Spearman’s rank correlation table of all differentially abundant taxa with social interaction score, neuroinflammatory and morphometric parameters, and TJ markers in male offspring; Table S6: Spearman’s rank correlation table of all differentially abundant taxa with social interaction score, neuroinflammatory and morphometric parameters, and TJ markers in female offspring.
