# Supplementary material for: Prenatal Stress Rewires the Gut–Brain Axis: Long-Term, Sex-Specific Effects on Microbiota, Intestinal Barrier, and Hippocampal Inflammation
Source: Nutrients. 2025 Aug 29;17(17):2812. doi: 10.3390/nu17172812 (PMC12429922; doi:10.3390/nu17172812)
Supplement: Supplementary file 1 [file nutrients-17-02812-s001.zip › nutrients-3833295-supplementary.pdf]

SUPPLEMENTARY MATERIALS

Prenatal Stress Rewires the Gut–Brain Axis: Long-Term, Sex-Specific Effects on Microbiota, Intestinal Barrier, and Hippocampal Inflammation †

Floriana De Cillis[1], Giulia Petrillo[1], Ilari D'Aprile[1], Moira Marizzoni[2], Samantha Saleri[2], Monica Mazzelli[2], Valentina Zonca[1], Maria Grazia Di Benedetto[2], Marco Andrea Riva[1,2], Annamaria Cattaneo[1,2*]

[1] Department of Pharmacological and Biomolecular Sciences, University of Milan, Milan, Italy
[2] Biological Psychiatry Laboratory, IRCCS Istituto Centro San Giovanni di Dio Fatebenefratelli, Brescia, Italy
* Correspondence: acattaneo@fatebenefratelli.eu; annamaria.cattaneo@unimi.it

This article is a revised and expanded version of a conference abstract entitled "Long-term effect of prenatal stress on offspring gut microbiome composition and functionality: possible implications on behavioural outcomes", which was presented at the 53rd Annual Meeting of the ISPNE, 29 -31 August and 1 September 2023, London, UK.

MATERIALS AND METHODS

Experimental design

The sample size of the behavioural test was already described in our previous study (D'Aprile & Petrillo, 2025). Specifically, behavioural assessment of male offspring included 34 CTRL animals and 32 PNS animals. The SI test was used to segregate the animals in RES and VULN. The cut-off value (mean of CTRL group measures minus one standard deviation) segregates the PNS group into 17 RES and 9 VULN animals. Behavioural assessment of female offspring included 34 CTRL animals and 32 PNS animals. The cut-off value (mean of CTRL group measures minus one standard deviation) segregated the PNS group into 21 RES and 11 VULN animals.

Gene expression analysis of neuroinflammatory markers in the DH of male animals included 22 CTRL and 23 PNS samples. The sub-groups consisted of 14 RES and 9 VULN animals. For females, gene expression analyses included 22 CTRL and 29 PNS samples, with sub-groups consisting of 18 RES and 11 VULN animals. Gene expression analysis of TJs included 12 CTRL and 12 PNS samples for both males and females. The sub-groups consisted of 4 RES and 8 VULN males, and 3 RES and 9 VULN females. Morphological and immunohistochemical analyses of males included 5 CTRL and 9 PNS samples, with sub-groups of 4 RES and 5 VULN. Morphological and immunohistochemical analyses of females included 5 CTRL and 8 PNS samples, with sub-groups of 3 RES and 5 VULN. Analyses of crypt-associated microbiota of male offspring included 12 CTRL and 12 PNS samples, with sub-groups of 4 RES and 8 VULN. Analyses of luminal content in males included 10 CTRL and 10 PNS samples, with sub-groups of 3 RES and 7 VULN. Analyses of crypt microbiota of female offspring included 11 CTRL and 12 PNS samples, with sub-groups of 3 RES and 9 VULN. Analyses of luminal content in females included 12 CTRL and 11 PNS samples, with sub-groups of 3 RES and 8 VULN.

Supplementary Table S1. Primers and probe sequences

Gene	Sequence primer forward	Sequence primer reverse	Sequence probe
CD68	TTACGGACAGCTTACCTTTGG	CTTGAAGAGATGAATTCTGCGC	CAAACAGGACCGACATCAGAGCCA
CX3CR1	TTCCCTAGTTGTGGCATGAAG	ACCTTCTGAACTTTTCCCCAG	TTAGTGTGACGGAGACAGTGGCG

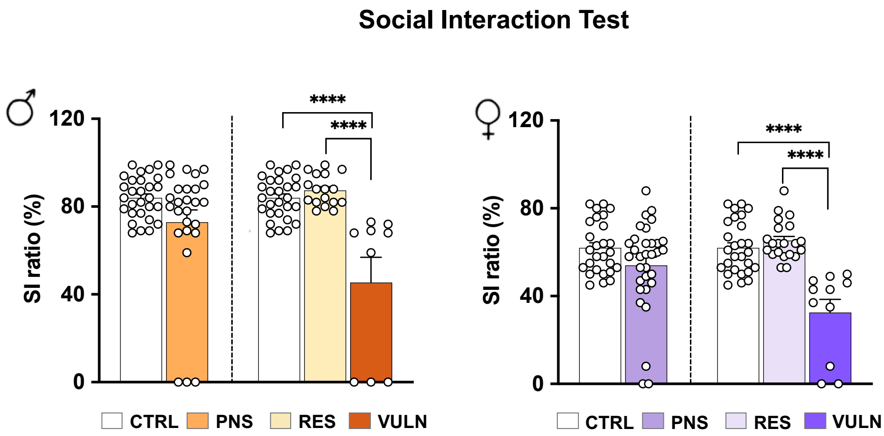

Supplementary Figure S1. Prenatal stress affects sociability. Sociability assessed through the social interaction ratio in adult male (left) and female (right) offspring. Sociability data were used to determine a cut-off (mean of CTRL group minus one standard deviation), enabling the segregation of the PNS group into RES and VULN. (****p < 0.0001). (Figure adapted from D'Aprile & Petrillo et al., 2025).

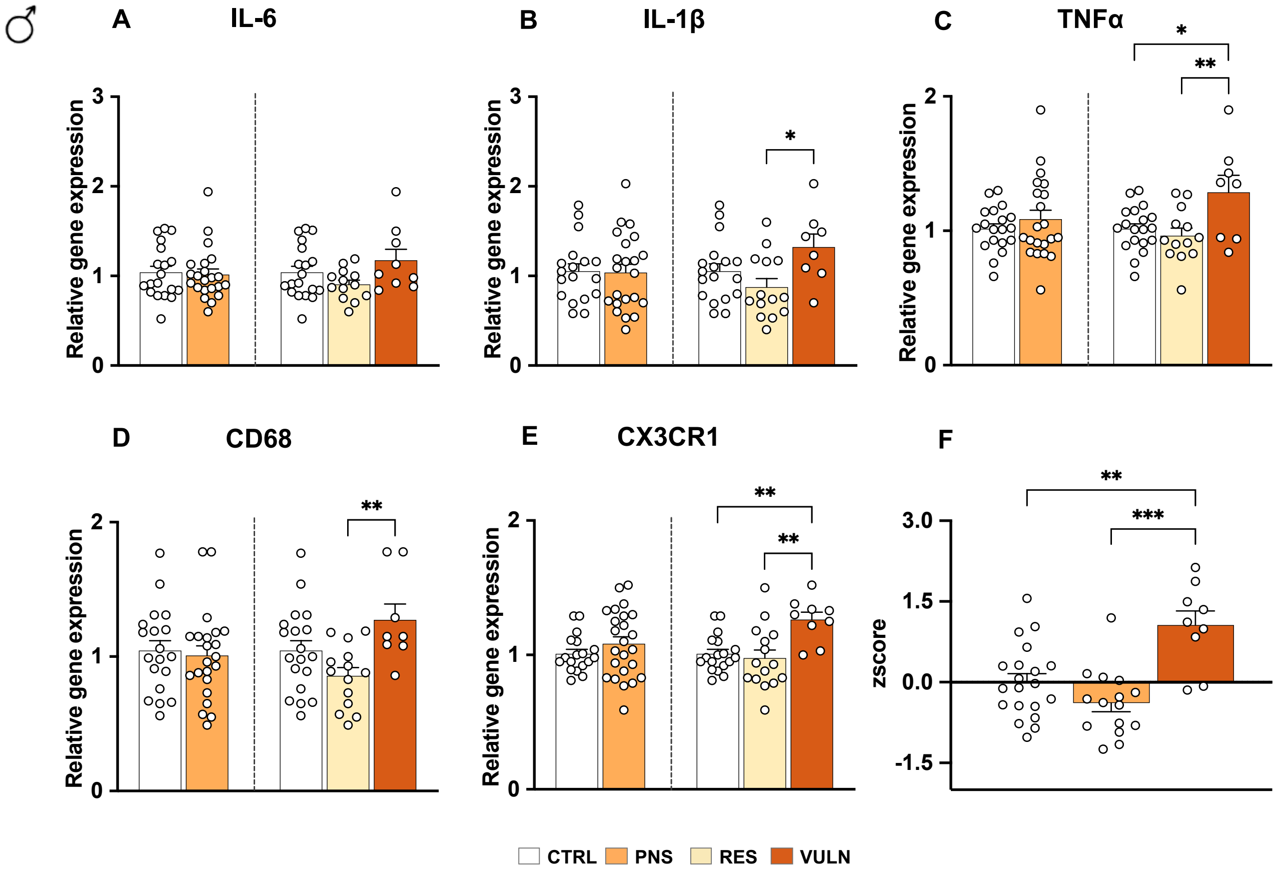

Supplementary Figure S2. Gene expression analysis of inflammation-related and microglia activation markers in the ventral hippocampus of male offspring. (A-E) Gene expression of IL-6, IL-1β, TNFα, CD68 and CX3CR1 assessed by qPCR. In each graph, the left bar plots represent the CTRL and PNS comparison. The right bar plots represent the CTRL, RES and VULN comparison. (F) The inflammatory z-score was calculated using the mRNA expression levels of the genes mentioned above, considering the CTRL, RES and VULN. Data are expressed as mean ± SEM in bar plots. (*p < 0.05, **p < 0.01, ***p < 0.001). (Figure adapted from D'Aprile & Petrillo et al., 2025).

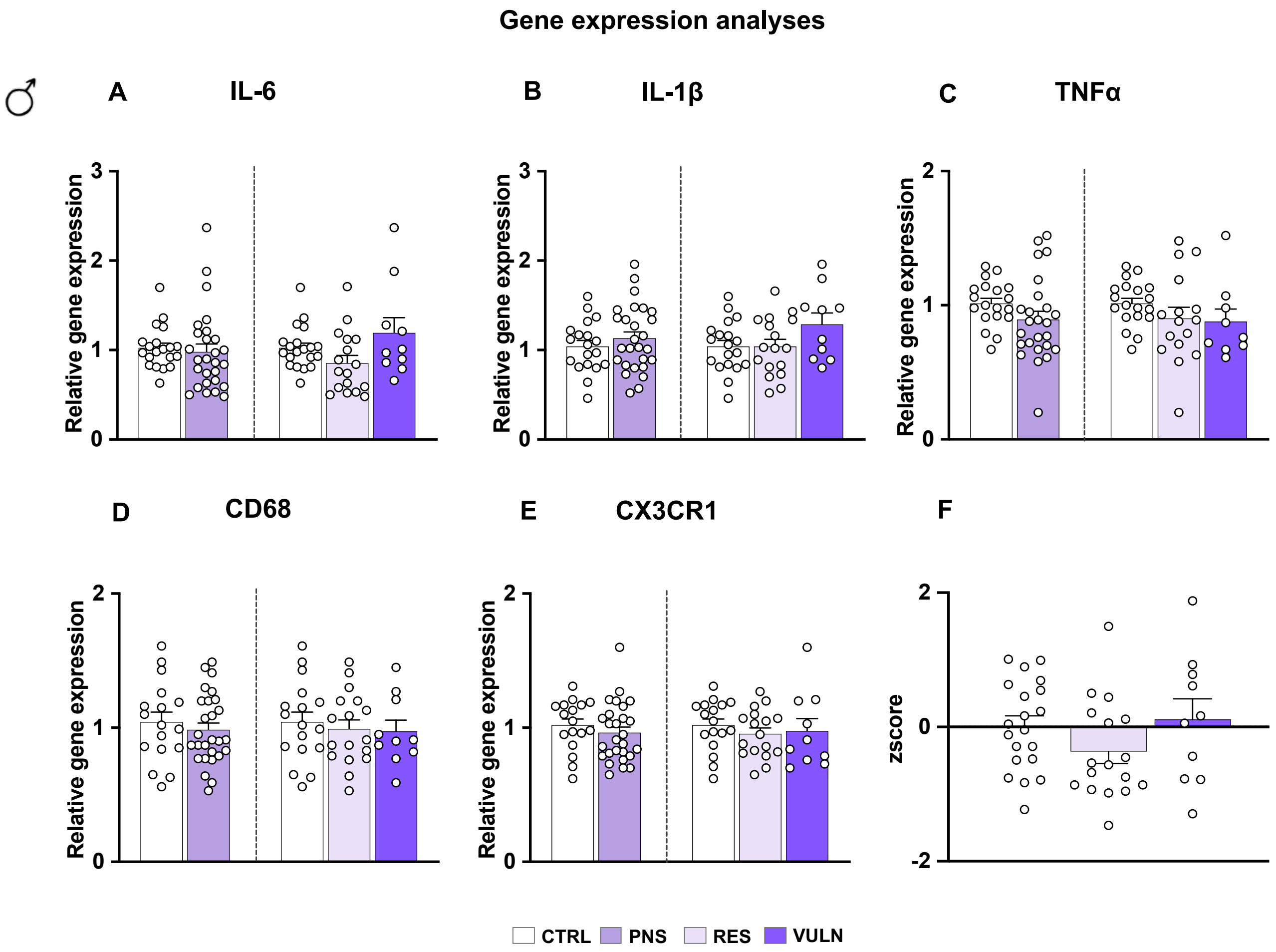

Supplementary Figure S 3. Gene expression analysis of inflammation-related and microglia activation markers in the ventral hippocampus of female offspring. (A-E) Gene expression of IL-6, IL-1β, TNFα, CD68 and CX3CR1 assessed by qPCR. In each graph, the left bar plots represent the CTRL and PNS comparison. The right bar plots represent the CTRL, RES and VULN comparison. (F) The inflammatory z-score was calculated using the mRNA expression levels of the genes mentioned above, taking into account the CTRL, RES and VULN. Data are expressed as mean ± SEM in bar plots. (Figure adapted and integrated from D'Aprile & Petrillo et al., 2025).

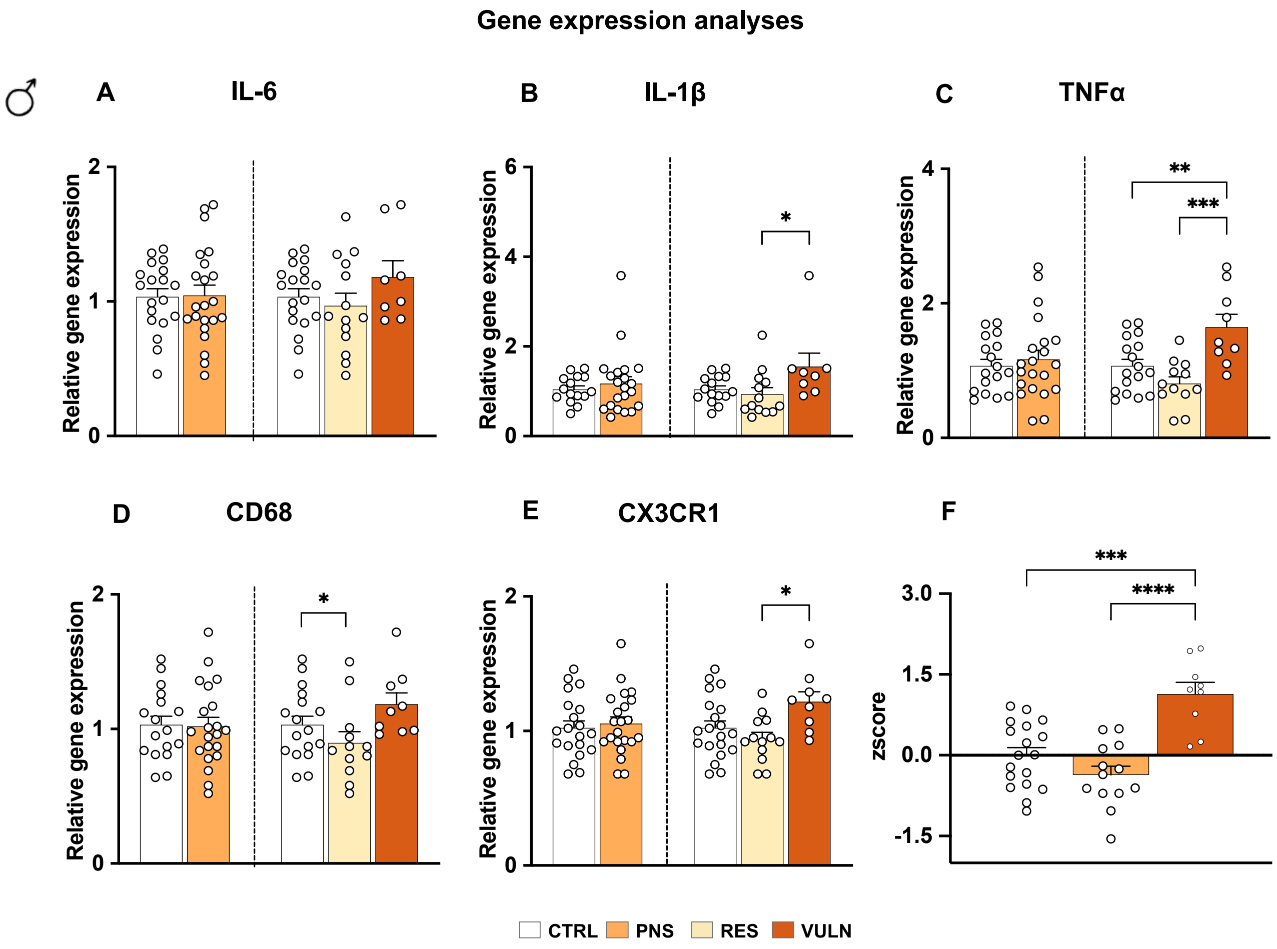

Supplementary Figure S4. Gene expression analysis of inflammation-related and microglia activation markers in the dorsal hippocampus of male offspring. (A-E) Gene expression of IL-6, IL-1β, TNFα, CD68 and CX3CR1 assessed by qPCR. In each graph, the left bar plots represent the CTRL and PNS comparison. The right bar plots represent the CTRL, RES and VULN comparison. (F) The inflammatory z-score was calculated using the mRNA expression levels of the genes mentioned above, taking into account the CTRL, RES and VULN. Data are expressed as mean ± SEM in bar plots. (*$p < 0.05$, **$p < 0.01$, ***$p < 0.001$).

Gene expression analyses

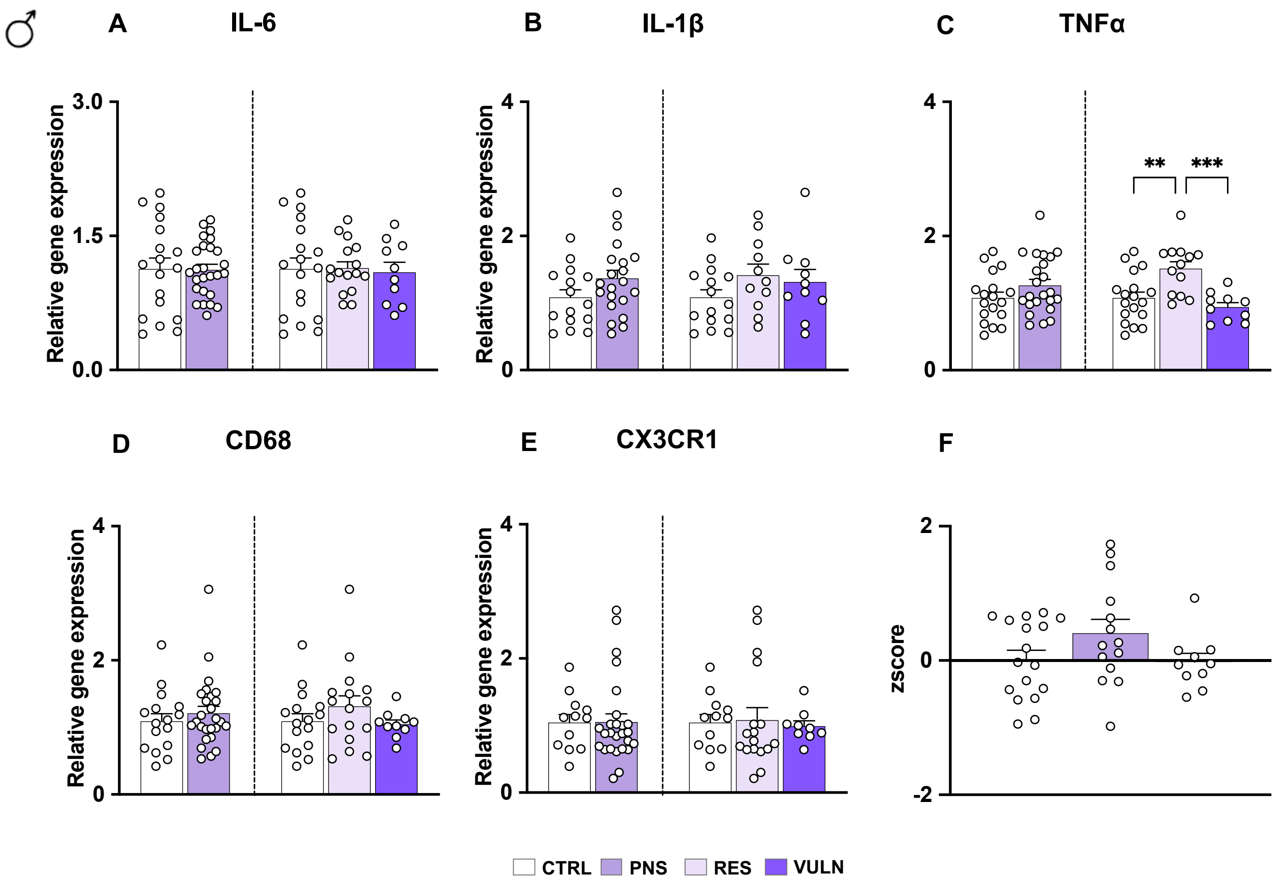

Supplementary Figure S5. Gene expression analysis of microglia activation and inflammation-related markers in the dorsal hippocampus of female offspring. (A-E) Gene expression of IL-6, IL-1β, TNFα, CD68 and CX3CR1 assessed by qPCR. In each graph, the left bar plots represent the CTRL and PNS comparison. The right bar plots represent the CTRL, RES and VULN comparison. (F) The inflammatory z-score was calculated using the mRNA expression levels of the genes mentioned above, taking into account the CTRL, RES and VULN. Data are expressed as mean ± SEM in bar plots. (**$p < 0.01$, ***$p < 0.001$).

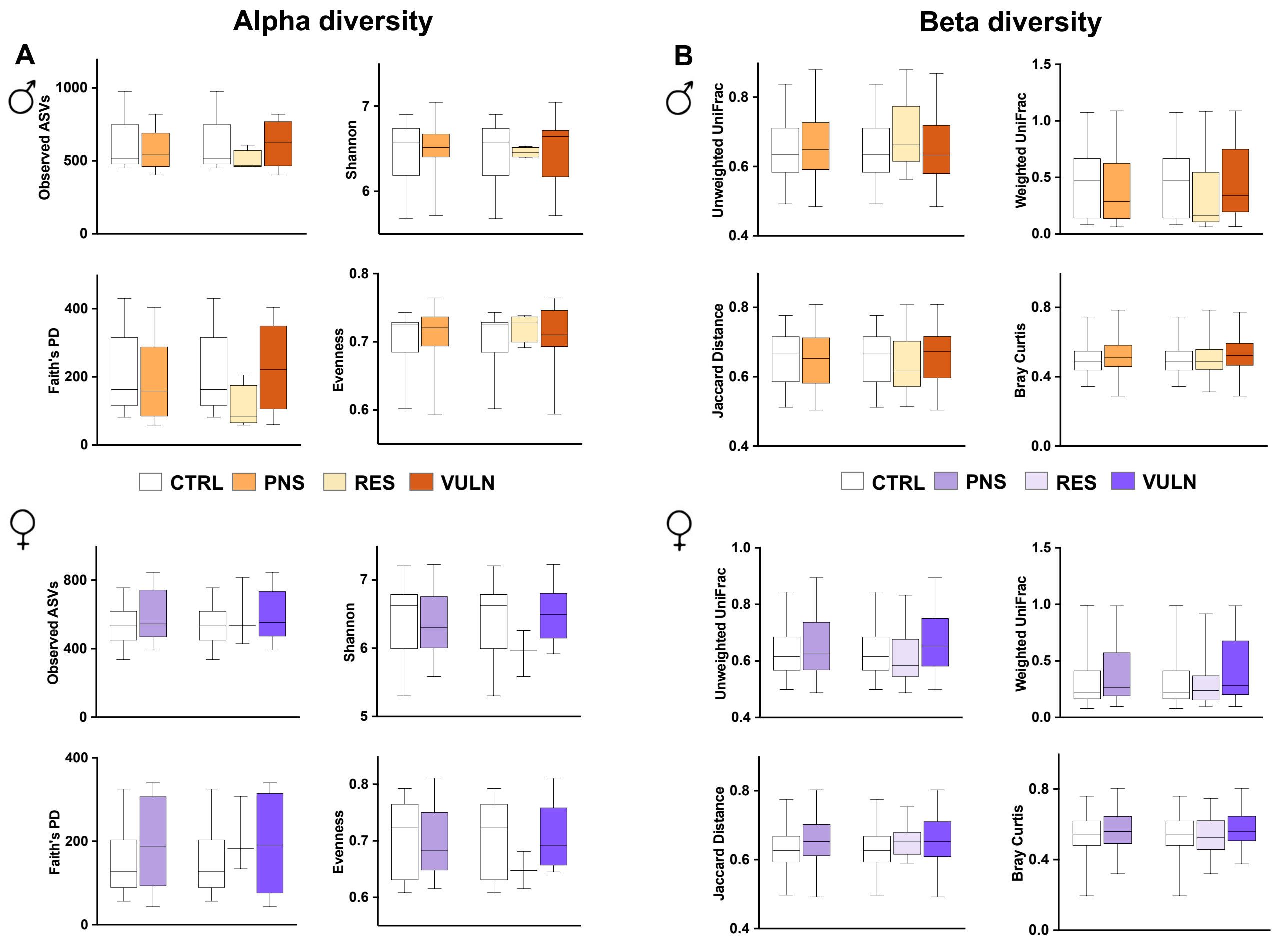

Supplementary Figure S6. Analysis of alpha and beta diversity of the crypt microbiota in male and female offspring. (A) Boxplots showing alpha diversity metrics Observed ASVs, Shannon index, Faith's Phylogenetic Diversity, and Evenness for male (top) and female (bottom) offspring. (B) Boxplots depicting beta diversity metrics Unweighted UniFrac, Weighted UniFrac, Jaccard distance, and Bray–Curtis dissimilarity for male (top) and female (bottom) offspring. In each graph, the left bar plots represent the CTRL and PNS comparison. The right bar plots represent the CTRL, RES and VULN comparison.

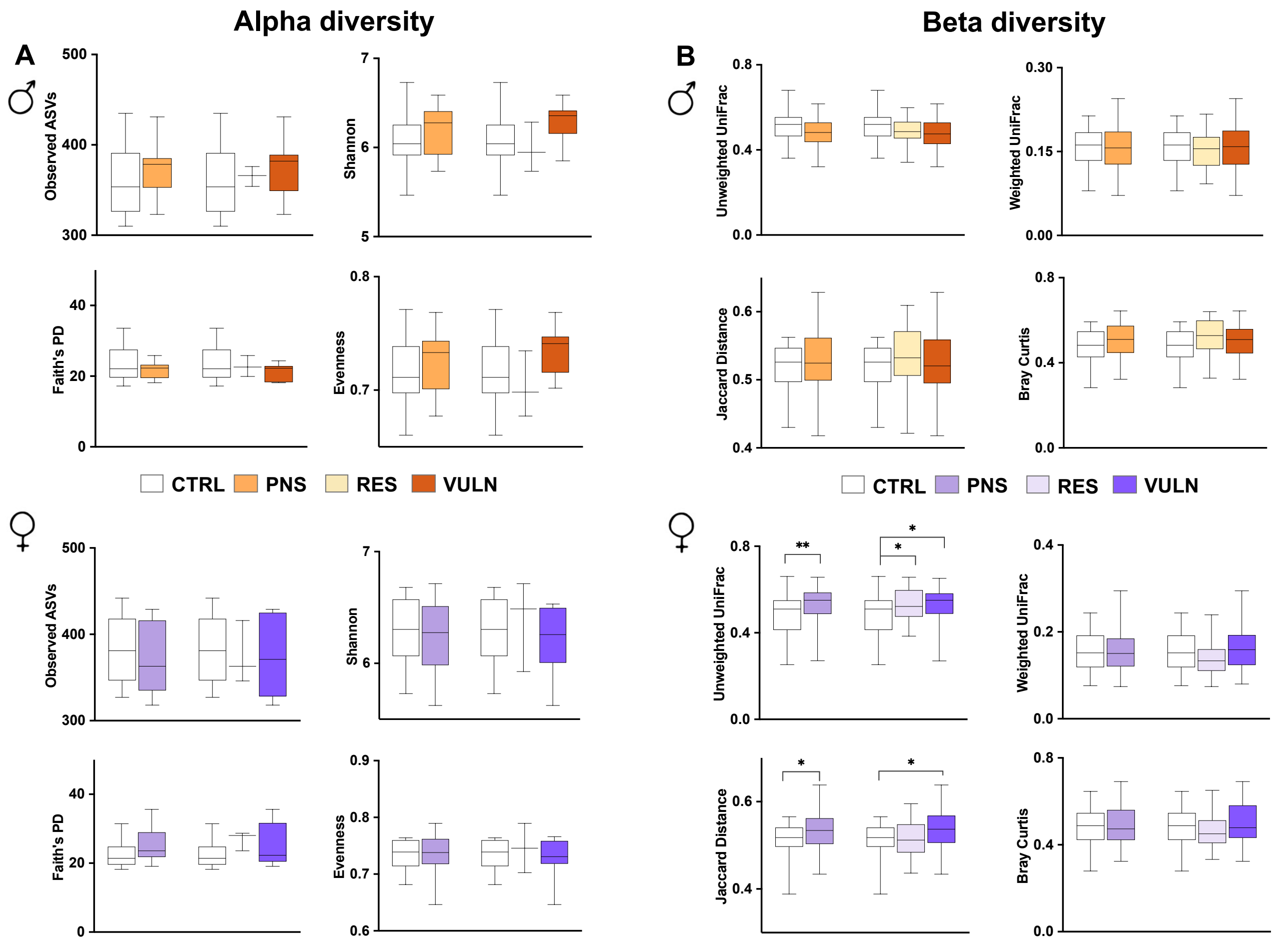

Supplementary Figure S7. Analysis of alpha and beta diversity of the luminal content in male and female offspring. (A) Boxplots showing alpha diversity metrics Observed ASVs, Shannon index, Faith's Phylogenetic Diversity, and Evenness for male (top) and female (bottom) offspring. (B) Boxplots depicting beta diversity metrics Unweighted UniFrac, Weighted UniFrac, Jaccard distance, and Bray–Curtis dissimilarity for male (top) and female (bottom) offspring. In each graph, the left bar plots represent the CTRL and PNS comparison. The right bar plots represent the CTRL, RES and VULN comparison. (*$p < 0.05$, **$p < 0.01$).

Crypt microbiota in Male offspring					
**PNSvsCTRL comparison**	**value**	**coef**	**stderr**	**pval**	**qval**
**p__Verrucomicrobiota**	PNS	-4,523	0,949	< 0,001	0,001
**p__Patescibacteria**	PNS	1,803	0,831	0,041	0,144
**RES&VULNvsCTRL comparison**					
**d__Bacteria_p__Verrucomicrobiota**	VULN	-4,391	1,085	0,001	0,008
**d__Bacteria_p__Verrucomicrobiota**	RES	-4,787	1,372	0,002	0,015
**d__Bacteria_p__Patescibacteria**	VULN	2,345	0,913	0,018	0,084
**Luminal content in Male offspring**					
**PNSvsCTRL comparison**					
**d__Bacteria_p__Verrucomicrobiota**	PNS	-3,120	0,768	0,001	0,005
**d__Bacteria_p__Patescibacteria**	PNS	2,937	0,832	0,002	0,008
**RES&VULNvsCTRL comparison**					
**d__Bacteria_p__Patescibacteria**	VULN	3,478	0,891	0,001	0,016
**d__Bacteria_p__Verrucomicrobiota**	VULN	-2,913	0,862	0,004	0,025
**d__Bacteria_p__Verrucomicrobiota**	RES	-3,603	1,152	0,006	0,029
**Luminal content in Female offspring**					
**PNSvsCTRL comparison**					
**d__Bacteria_p__Desulfobacterota**	PNS	1,745	0,692	0,020	0,069
**d__Bacteria_p__Patescibacteria**	PNS	-1,806	0,681	0,015	0,069
**RES&VULNvsCTRL comparison**					
**d__Bacteria_p__Desulfobacterota**	VULN	2,040	0,758	0,014	0,098
**d__Bacteria_p__Patescibacteria**	VULN	-2,119	0,744	0,010	0,098

Supplementary Table S2. Significant phylum-level differences in crypt and lumen by group and sex. MaAsLin analysis results of significant associations at phylum level in the crypt and luminal content analyses, comparing the whole group of PNS than CTRL and the sub-group of RES and VULN animals, in male and female offspring.

Crypt microbiota in Male offspring					
**Features - PNSvsCTRL comparison**	value	coef	stderr	pval	qval
p__Verrucomicrobiota.c__Verrucomicrobiae.o__Verrucomicrobiales.f__Akkermansiaceae.g__Akkermansia	PNS	-5,125	1,004	< 0,001	0,005
p__Firmicutes.c__Clostridia.o__Peptostreptococcales.Tissierellales.f__Anaerovoracaceae.g__Anaerovorax	PNS	1,657	0,636	0,018	0,411
p__Actinobacteriota.c__Actinobacteria.o__bacteriales.f__Corynebacteriaceae.g__Corynebacterium	PNS	2,020	0,689	0,008	0,219
p__Firmicutes.c__Clostridia.o__Eubacteriales.f__Anaerofustaceae.g__Anaerofustis	PNS	1,485	0,499	0,007	0,219
p__Actinobacteriota.c__Actinobacteria.o__Propionibacteriales.f__Propionibacteriaceae.g__Cutibacterium	PNS	-1,696	0,620	0,012	0,275
p__Firmicutes.c__Clostridia.o__Oscillospirales.f__Ruminococcaceae.g__Anaerotruncus	PNS	1,382	0,626	0,038	0,551
p__Patescibacteria.c__Saccharimonadia.o__Saccharimonadales.f__Saccharimonadaceae.g__Candidatus_Saccharimonas	PNS	1,390	0,644	0,042	0,551
p__Firmicutes.c__Clostridia.o__Lachnospirales.f__Lachnospiraceae.g__Lachnospiraceae UCG 008	PNS	-0,613	0,282	0,041	0,551
p__Firmicutes.c__Bacilli.o__Staphylococcales.f__Staphylococcaceae.g__Auricoccus.Abyssicoccus	PNS	1,256	0,589	0,044	0,551
p__Firmicutes.c__Clostridia.o__Lachnospirales.f__Lachnospiraceae.g__Anaerostipes	PNS	-0,974	0,467	0,049	0,551
**Features - RES&VULNvsCTRL comparison**					
p__Verrucomicrobiota.c__Verrucomicrobiae.o__Verrucomicrobiales.f__Akkermansiaceae.g__Akkermansia	VULN	-5,029	1,148	< 0,001	0,059
p__Verrucomicrobiota.c__Verrucomicrobiae.o__Verrucomicrobiales.f__Akkermansiaceae.g__Akkermansia	RES	-5,318	1,452	0,001	0,164
p__Actinobacteriota.c__Actinobacteria.o__bacteriales.f__Corynebacteriaceae.g__Corynebacterium	RES	3,105	0,940	0,003	0,223
p__Firmicutes.c__Clostridia.o__Peptostreptococcales.Tissierellales.f__Anaerovoracaceae.g__Anaerovorax	VULN	1,910	0,732	0,016	0,528
p__Firmicutes.c__Bacilli.o__Erysipelotrichales.f__Erysipelotrichaceae.g__Allobaculum	VULN	-1,611	0,588	0,012	0,462
p__Firmicutes.c__Clostridia.o__Eubacteriales.f__Anaerofustaceae.g__Anaerofustis	VULN	1,575	0,569	0,012	0,462
p__Firmicutes.c__Clostridia.o__Oscillospirales.f__Oscillospiraceae.g__Intestinimonas	RES	1,532	0,629	0,024	0,563
p__Patescibacteria.c__Saccharimonadia.o__Saccharimonadales.f__Saccharimonadaceae.g__Candidatus_Saccharimonas	VULN	1,734	0,718	0,025	0,563
p__Firmicutes.c__Bacilli.o__Staphylococcales.f__Staphylococcaceae.g__Auricoccus.Abyssicoccus	VULN	1,601	0,652	0,023	0,563
p__Firmicutes.c__Clostridia.o__Clostridiales.f__Clostridiaceae.g__Clostridium_sensu_stricto_1	VULN	-3,189	1,406	0,034	0,637
p__Actinobacteriota.c__Actinobacteria.o__Propionibacteriales.f__Propionibacteriaceae.g__Cutibacterium	VULN	-1,551	0,707	0,039	0,637
p__Actinobacteriota.c__Actinobacteria.o__Propionibacteriales.f__Propionibacteriaceae.g__Cutibacterium	RES	-1,984	0,894	0,038	0,637
p__Bacteroidota.c__Bacteroidia.o__Bacteroidales.f__Bacteroidaceae.g__Bacteroides	VULN	-0,583	0,280	0,050	0,752
**Features - RESvsVULN comparison**					
p__Firmicutes.c__Bacilli.o__Erysipelotrichales.f__Erysipelotrichaceae.g__Allobaculum	RES	2,703	0,789	0,003	0,245
p__Firmicutes.c__Clostridia.o__Oscillospirales.f__Oscillospiraceae.g__Intestinimonas	RES	1,584	0,668	0,027	0,644
**Luminal content in Male offspring**					
**Features - PNSvsCTRL comparison**					
p__Actinobacteriota.c__Actinobacteria.o__Corynebacteriales.f__Corynebacteriaceae.g__Corynebacterium	PNS	2,094	0,536	0,001	0,053
p__Verrucomicrobiota.c__Verrucomicrobiae.o__Verrucomicrobiales.f__Akkermansiaceae.g__Akkermansia	PNS	-3,114	0,767	0,001	0,053
p__Patescibacteria.c__Saccharimonadia.o__Saccharimonadales.f__Saccharimonadaceae.g__Candidatus_Saccharimonas	PNS	2,843	0,905	0,006	0,188
p__Firmicutes.c__Clostridia.o__Lachnospirales.f__Lachnospiraceae.g__Anaerostipes	PNS	-3,429	1,133	0,007	0,188
p__Firmicutes.c__Clostridia.o__Clostridiales.f__Clostridiaceae.g__Clostridium_sensu_stricto_1	PNS	-2,993	1,053	0,011	0,212
p__Firmicutes.c__Clostridia.o__Monoglobales.f__Monoglobaceae.g__Monoglobus	PNS	2,502	1,001	0,022	0,258
p__Firmicutes.c__Clostridia.o__Lachnospirales.f__Defluviitaleaceae.g__Defluviitaleaceae UCG 011	PNS	1,080	0,432	0,022	0,258
p__Firmicutes.c__Clostridia.o__Oscillospirales.f__Eubacterium_coprostanoligenes_group.g__Eubacterium_coprostanoligenes_group	PNS	1,035	0,431	0,028	0,286
p__Firmicutes.c__Clostridia.o__Peptostreptococcales.Tissierellales.f__Anaerovoracaceae.g__Eubacterium_brachy_group	PNS	0,817	0,366	0,038	0,291
p__Firmicutes.c__Bacilli.o__Lactobacillales.f__Streptococcaceae.g__Streptococcus	PNS	1,275	0,570	0,038	0,291
p__Firmicutes.c__Clostridia.o__Lachnospirales.f__Lachnospiraceae.g__Lachnospiraceae_FCS020_group	PNS	1,829	0,822	0,039	0,291
p__Firmicutes,c__Bacilli,o__Erysipelotrichales,f__Erysipelotrichaceae,g__Allobaculum	PNS	-0,849	0,391	0,043	0,301
**Features - RES&VULNvsCTRL comparison**					
p__Firmicutes.c__Clostridia.o__Oscillospirales.f__Oscillospiraceae.g__Oscillospira	RES	-3,132	0,751	0,001	0,133
p__Actinobacteriota.c__Actinobacteria.o__Corynebacteriales.f__Corynebacteriaceae.g__Corynebacterium	VULN	2,064	0,607	0,003	0,177
p__Verrucomicrobiota.c__Verrucomicrobiae.o__Verrucomicrobiales.f__Akkermansiaceae.g__Akkermansia	VULN	-2,901	0,861	0,004	0,177
p__Patescibacteria.c__Saccharimonadia.o__Saccharimonadales.f__Saccharimonadaceae.g__Candidatus_Saccharimonas	VULN	3,322	0,988	0,004	0,177
p__Firmicutes.c__Clostridia.o__Oscillospirales.f__Oscillospiraceae.g__Colidextribacter	RES	-2,321	0,722	0,005	0,177
p__Firmicutes.c__Clostridia.o__Lachnospirales.f__Defluviitaleaceae.g__Defluviitaleaceae UCG 011	RES	1,900	0,596	0,005	0,177
p__Verrucomicrobiota.c__Verrucomicrobiae.o__Verrucomicrobiales.f__Akkermansiaceae.g__Akkermansia	RES	-3,611	1,150	0,006	0,177
p__Firmicutes.c__Clostridia.o__Oscillospirales.f__Eubacterium_coprostanoligenes_group.g__Eubacterium_coprostanoligenes_group	RES	1,834	0,598	0,007	0,181
p__Firmicutes.c__Clostridia.o__Monoglobales.f__Monoglobaceae.g__Monoglobus	VULN	3,045	1,091	0,013	0,260
p__Bacteroidota.c__Bacteroidia.o__Bacteroidales.f__Marinifilaceae.g__Odoribacter	VULN	0,761	0,280	0,015	0,260
p__Firmicutes.c__Clostridia.o__Lachnospirales.f__Lachnospiraceae.g__Anaerostipes	VULN	-3,429	1,284	0,016	0,260
p__Actinobacteriota.c__Actinobacteria.o__Corynebacteriales.f__Corynebacteriaceae.g__Corynebacterium	RES	2,163	0,811	0,016	0,260
p__Firmicutes.c__Clostridia.o__Clostridiales.f__Clostridiaceae.g__Clostridium_sensu_stricto_1	VULN	-2,934	1,193	0,025	0,337
p__Firmicutes.c__Clostridia.o__Oscillospirales.f__Butyricicoccaceae.g__UCG 009	VULN	1,263	0,518	0,026	0,337
p__Firmicutes.c__Clostridia.o__Oscillospirales.f__Oscillospiraceae.g__UCG 005	VULN	-1,662	0,691	0,028	0,340
p__Firmicutes.c__Clostridia.o__Peptostreptococcales.Tissierellales.f__Anaerovoracaceae.g__Family_XIII_UCG 001	RES	-2,205	0,970	0,036	0,397
**Features - RESvsVULN comparison**					
p__Firmicutes.c__Clostridia.o__Oscillospirales.f__Oscillospiraceae.g__Oscillospira	RES	-2,824	0,787	0,002	0,192
p__Firmicutes.c__Clostridia.o__Oscillospirales.f__Oscillospiraceae.g__Colidextribacter	RES	-2,239	0,756	0,009	0,304
p__Firmicutes.c__Clostridia.o__Oscillospirales.f__Butyricicoccaceae.g__UCG 009	RES	-1,910	0,725	0,017	0,329
p__Bacteroidota.c__Bacteroidia.o__Bacteroidales.f__Marinifilaceae.g__Odoribacter	RES	-0,961	0,393	0,025	0,360
p__Firmicutes.c__Clostridia.o__Oscillospirales.f__Ruminococcaceae.g__UBA1819	RES	-1,516	0,621	0,026	0,360
p__Firmicutes.c__Clostridia.o__Peptostreptococcales.Tissierellales.f__Anaerovoracaceae.g__Family_XIII_UCG 001	RES	-2,248	1,017	0,041	0,474

Supplementary Table S3. Significant genus-level microbial differences in the crypt and lumen of male offspring. MaAsLin analysis results of significant associations at the genus level in the crypt and luminal content analyses, comparing the whole group of PNS than CTRL and the sub-group of RES and VULN animals, in male offspring.

Crypt microbiota in Female offspring					
**Features - PNSvsCTRL comparison**	value	coef	stderr	pval	qval
p__Firmicutes.c__Clostridia.o__Lachnospirales.f__Lachnospiraceae.g__Syntrophococcus	PNS	-1,791	0,552	0,004	0,348
p__Firmicutes.c__Clostridia.o__Oscillospirales.f__Ruminococcaceae.g__Paludicola	PNS	1,379	0,473	0,008	0,348
p__Firmicutes.c__Clostridia.o__Oscillospirales.f__UCG 010.g__UCG 010	PNS	-2,097	0,752	0,011	0,348
p__Firmicutes.c__Bacilli.o__Erysipelotrichales.f__Erysipelatoclostridiaceae.g__Erysipelatoclostridium	PNS	1,388	0,523	0,015	0,348
p__Firmicutes.c__Clostridia.o__Lachnospirales.f__Lachnospiraceae.g__Tuzzerella	PNS	-1,538	0,587	0,016	0,348
p__Firmicutes.c__Clostridia.o__Oscillospirales.f__Butyricicoccaceae.g__UCG 009	PNS	-1,379	0,557	0,022	0,386
p__Firmicutes.c__Clostridia.o__Peptostreptococcales.Tissierellales.f__Anaerovoracaceae.g__Eubacterium_brachy_group	PNS	1,302	0,538	0,025	0,386
p__Firmicutes.c__Clostridia.o__Clostridia_vadinBB60_group.f__Clostridia_vadinBB60_group.g__Clostridia_vadinBB60_group	PNS	-1,254	0,549	0,033	0,408
p__Firmicutes.c__Clostridia.o__Oscillospirales.f__Ruminococcaceae.g__Eubacterium_siraeum_group	PNS	-0,598	0,269	0,037	0,408
p__Firmicutes.c__Clostridia.o__Lachnospirales.f__Lachnospiraceae.g__Roseburia	PNS	-1,994	0,907	0,039	0,408
d__Bacteria.p__Patescibacteria.c__Saccharimonadia.o__Saccharimonadales.f__Saccharimonadaceae.g__Candidatus_Saccharimonas	PNS	-0,876	0,397	0,039	0,381
p__Firmicutes.c__Clostridia.o__Monoglobales.f__Monoglobaceae.g__Monoglobus	PNS	1,731	0,806	0,044	0,408
p__Firmicutes.c__Clostridia.o__Lachnospirales.f__Lachnospiraceae.g__Lachnospiraceae UCG 008	PNS	0,544	0,255	0,045	0,408
p__Firmicutes.c__Clostridia.o__Clostridiales.f__Clostridiaceae.g__Clostridium_sensu_stricto_1	PNS	-3,188	1,535	0,050	0,422
**Features - RES&VULNvsCTRL comparison**					
p__Firmicutes.c__Clostridia.o__Clostridia_vadinBB60_group.f__Clostridia_vadinBB60_group.g__Clostridia_vadinBB60_group	VULN	-1,748	0,527	0,003	0,520
p__Firmicutes.c__Clostridia.o__Oscillospirales.f__Ruminococcaceae.g__Paludicola	VULN	1,519	0,515	0,008	0,520
p__Firmicutes.c__Clostridia.o__Lachnospirales.f__Lachnospiraceae.g__Syntrophococcus	VULN	-1,791	0,610	0,008	0,520
p__Firmicutes.c__Clostridia.o__Lachnospirales.f__Lachnospiraceae.g__Tuzzerella	VULN	-1,790	0,629	0,010	0,520
p__Firmicutes.c__Clostridia.o__Peptostreptococcales.Tissierellales.f__Anaerovoracaceae.g__Eubacterium_brachy_group	VULN	1,577	0,570	0,012	0,520
p__Firmicutes.c__Clostridia.o__Lachnospirales.f__Lachnospiraceae.g__Roseburia	VULN	-2,460	0,960	0,019	0,521
p__Firmicutes.c__Bacilli.o__Erysipelotrichales.f__Erysipelatoclostridiaceae.g__Erysipelatoclostridium	RES	2,043	0,814	0,021	0,521
p__Firmicutes.c__Bacilli.o__RF39.f__RF39.g__RF39	RES	1,020	0,431	0,028	0,521
p__Firmicutes.c__Clostridia.o__Oscillospirales.f__UCG 010.g__UCG 010	VULN	-1,906	0,821	0,031	0,521
p__Firmicutes.c__Clostridia.o__Oscillospirales.f__UCG 010.g__UCG 010	RES	-2,672	1,190	0,036	0,521
p__Bacteroidota.c__Bacteroidia.o__Bacteroidales.f__Muribaculaceae.g__Muribaculum	RES	-1,993	0,891	0,037	0,521
p__Firmicutes.c__Clostridia.o__Monoglobales.f__Monoglobaceae.g__Monoglobus	RES	2,766	1,254	0,039	0,521
d__Bacteria.p__Patescibacteria.c__Saccharimonadia.o__Saccharimonadales.f__Saccharimonadaceae.g__Candidatus_Saccharimonas	VULN	-0,960	0,435	0,039	0,480
p__Firmicutes.c__Clostridia.o__Clostridiales.f__Clostridiaceae.g__Clostridium_sensu_stricto_1	RES	-5,210	2,383	0,041	0,521
p__Cyanobacteria.c__Cyanobacteriia.o__Chloroplast.f__Chloroplast.g__Chloroplast	RES	0,834	0,382	0,041	0,521
p__Firmicutes.c__Clostridia.o__Oscillospirales.f__Eubacterium_coprostanoligenes_group.g__Eubacterium_coprostanoligenes_group	RES	-1,401	0,661	0,047	0,521
p__Actinobacteriota.c__Coriobacteriia.o__Coriobacteriales.f__Eggerthellaceae.g__Enterorhabdus	VULN	0,813	0,387	0,048	0,521
**Features - RESvsVULN comparison**					
p__Bacteroidota.c__Bacteroidia.o__Bacteroidales.f__Muribaculaceae.g__Muribaculum	RES	-2,690	0,912	0,008	0,433
p__Firmicutes.c__Clostridia.o__Clostridia_vadinBB60_group.f__Clostridia_vadinBB60_group.g__Clostridia_vadinBB60_group	RES	1,977	0,782	0,020	0,544
p__Firmicutes.c__Clostridia.o__Oscillospirales.f__Eubacterium_coprostanoligenes_group.g__Eubacterium_coprostanoligenes_group	RES	-1,430	0,676	0,047	0,721
**Luminal content in Female offspring**					
**Features - PNSvsCTRL comparison**					
p__Firmicutes.c__Clostridia.o__Clostridiales.f__Clostridiaceae.g__Clostridium_sensu_stricto_1	PNS	-5,902	1,364	< 0,001	0,030
p__Firmicutes.c__Clostridia.o__Oscillospirales.f__Ruminococcaceae.g__Anaerotruncus	PNS	2,223	0,576	0,001	0,030
p__Firmicutes.c__Bacilli.o__Erysipelotrichales.f__Erysipelatoclostridiaceae.g__Erysipelatoclostridium	PNS	1,408	0,374	0,001	0,030
p__Proteobacteria.c__Gammaproteobacteria.o__Pasteurellales.f__Pasteurellaceae.g__Haemophilus	PNS	1,766	0,465	0,001	0,030
p__Actinobacteriota.c__Coriobacteriia.o__Coriobacteriales.f__Atopobiaceae.g__Coriobacteriaceae_UCG 002	PNS	1,596	0,500	0,004	0,093
p__Proteobacteria.c__Gammaproteobacteria.o__Enterobacterales.f__Enterobacteriaceae.g__Escherichia Shigella	PNS	-2,439	0,825	0,008	0,124
p__Firmicutes.c__Clostridia.o__Oscillospirales.f__Ruminococcaceae.g__Incertae_Sedis	PNS	0,918	0,320	0,009	0,124
p__Firmicutes.c__Clostridia.o__Oscillospirales.f__Oscillospiraceae.g__UCG 002	PNS	-0,928	0,331	0,011	0,124
p__Firmicutes.c__Clostridia.o__Lachnospirales.f__Defluviitaleaceae.g__Defluviitaleaceae UCG 011	PNS	1,303	0,464	0,011	0,124
p__Patescibacteria.c__Saccharimonadia.o__Saccharimonadales.f__Saccharimonadaceae.g__Candidatus_Saccharimonas	PNS	-1,832	0,696	0,016	0,165
p__Firmicutes.c__Clostridia.o__Lachnospirales.f__Lachnospiraceae.g__Lachnoclostridium	PNS	1,095	0,434	0,020	0,168
p__Desulfobacterota.c__Desulfovibrionia.o__Desulfovibrionales.f__Desulfovibrionaceae.g__Desulfovibrio	PNS	1,726	0,683	0,020	0,168
p__Firmicutes.c__Clostridia.o__Peptostreptococcales.Tissierellales.f__Anaerovoracaceae.g__Family_XIII_UCG 001	PNS	1,081	0,432	0,021	0,168
p__Firmicutes.c__Clostridia.o__Oscillospirales.f__Butyricicoccaceae.g__Butyricicoccus	PNS	0,929	0,407	0,033	0,226
p__Firmicutes.c__Clostridia.o__Lachnospirales.f__Lachnospiraceae.g__Anaerostipes	PNS	1,146	0,506	0,034	0,226
p__Firmicutes.c__Clostridia.o__Monoglobales.f__Monoglobaceae.g__Monoglobus	PNS	1,743	0,831	0,048	0,291
**Features - RES&VULNvsCTRL comparison**					
p__Actinobacteriota.c__Coriobacteriia.o__Coriobacteriales.f__Atopobiaceae.g__Coriobacteriaceae_UCG 002	VULN	2,195	0,452	< 0,001	0,020
p__Firmicutes.c__Clostridia.o__Oscillospirales.f__Ruminococcaceae.g__Anaerotruncus	VULN	2,570	0,617	< 0,001	0,050
p__Firmicutes.c__Clostridia.o__Clostridiales.f__Clostridiaceae.g__Clostridium_sensu_stricto_1	VULN	-5,883	1,529	0,001	0,071
p__Firmicutes.c__Bacilli.o__Erysipelotrichales.f__Erysipelatoclostridiaceae.g__Erysipelatoclostridium	RES	2,0439	0,5619	0,002	0,0750
p__Proteobacteria.c__Gammaproteobacteria.o__Pasteurellales.f__Pasteurellaceae.g__Haemophilus	VULN	1,868	0,518	0,002	0,075
p__Firmicutes.c__Bacilli.o__Erysipelotrichales.f__Erysipelatoclostridiaceae.g__Erysipelatoclostridium	VULN	1,169	0,397	0,008	0,195
p__Firmicutes.c__Clostridia.o__Lachnospirales.f__Lachnospiraceae.g__Lachnoclostridium	RES	1,877	0,648	0,009	0,195
p__Proteobacteria.c__Gammaproteobacteria.o__Enterobacterales.f__Enterobacteriaceae.g__Escherichia Shigella	RES	-3,541	1,266	0,011	0,195
p__Patescibacteria.c__Saccharimonadia.o__Saccharimonadales.f__Saccharimonadaceae.g__Candidatus_Saccharimonas	VULN	-2,137	0,762	0,011	0,195
p__Firmicutes.c__Clostridia.o__Clostridiales.f__Clostridiaceae.g__Clostridium_sensu_stricto_1	RES	-5,953	2,162	0,012	0,195
p__Desulfobacterota.c__Desulfovibrionia.o__Desulfovibrionales.f__Desulfovibrionaceae.g__Desulfovibrio	VULN	2,030	0,747	0,013	0,195
p__Firmicutes.c__Clostridia.o__Oscillospirales.f__Ruminococcaceae.g__Incertae_Sedis	VULN	0,962	0,358	0,014	0,195
p__Firmicutes.c__Clostridia.o__Peptostreptococcales.Tissierellales.f__Anaerovoracaceae.g__Family_XIII_UCG 001	VULN	1,276	0,472	0,014	0,195
p__Firmicutes.c__Clostridia.o__Lachnospirales.f__Lachnospiraceae.g__Ruminococcus._torques_group	VULN	0,398	0,149	0,015	0,195
p__Firmicutes.c__Clostridia.o__Lachnospirales.f__Lachnospiraceae.g__Marvinbryantia	VULN	-2,311	0,900	0,018	0,216
p__Firmicutes.c__Clostridia.o__Oscillospirales.f__Oscillospiraceae.g__UCG 002	VULN	-0,928	0,370	0,021	0,227
p__Firmicutes.c__Clostridia.o__Lachnospirales.f__Defluviitaleaceae.g__Defluviitaleaceae UCG 011	VULN	1,297	0,520	0,021	0,227
p__Firmicutes.c__Clostridia.o__Clostridia_vadinBB60_group.f__Clostridia_vadinBB60_group.g__Clostridia_vadinBB60_group	VULN	-1,556	0,639	0,024	0,234
p__Firmicutes.c__Clostridia.o__Lachnospirales.f__Lachnospiraceae.g__Anaerostipes	VULN	1,353	0,555	0,024	0,234
p__Firmicutes.c__Clostridia.o__Lachnospirales.f__Lachnospiraceae.g__Lachnospiraceae_UCG 006	VULN	1,219	0,535	0,034	0,296
p__Firmicutes.c__Clostridia.o__Oscillospirales.f__Clostridium_methylpentosum_group.g__Clostridium_methylpentosum_group	RES	-2,402	1,055	0,034	0,296
p__Proteobacteria.c__Gammaproteobacteria.o__Enterobacterales.f__Enterobacteriaceae.g__Escherichia Shigella	VULN	-2,027	0,895	0,035	0,296
**Features - CTRL&RESvsVULN comparison**					
p__Actinobacteriota.c__Coriobacteriia.o__Coriobacteriales.f__Atopobiaceae.g__Coriobacteriaceae_UCG 002	RES	-2,195	0,670	0,004	0,161
p__Bacteroidota.c__Bacteroidia.o__Bacteroidales.f__Muribaculaceae.g__Muribaculum	RES	-1,703	0,665	0,019	0,239
p__Firmicutes.c__Clostridia.o__Lachnospirales.f__Lachnospiraceae.g__Marvinbryantia	RES	3,363	1,335	0,020	0,239
p__Firmicutes.c__Clostridia.o__Clostridia_vadinBB60_group.f__Clostridia_vadinBB60_group.g__Clostridia_vadinBB60_group	RES	2,156	0,947	0,034	0,308
p__Firmicutes.c__Clostridia.o__Oscillospirales.f__Clostridium_methylpentosum_group.g__Clostridium_methylpentosum_group	RES	-2,483	1,107	0,036	0,308

Supplementary Table S4. Significant genus-level microbial differences in the crypt and lumen of female offspring**.** MaAsLin analysis results of significant associations at the genus level in the crypt and luminal content analyses, comparing the whole group of PNS than CTRL and the sub-group of RES and VULN animals, in female offspring.

		Ventral Hippocampus						Dorsal Hippocampus										
**Crypt microbiota in Male offspring**	**SI score**	**IL-6**	**IL-1β**	**TNFα**	**CX3CR1**	**CD68**	**z-score VH**	**IL-6**	**IL-1β**	**TNFα**	**CX3CR1**	**CD68**	**z-score DH**	**Villus lenght**	**Crypt lenght**	**Surface Area**	**Ocln**	**Z0-1**
**Akkermansia**	0,599*	-0,320	-0,365	-0,265	-0,530	0,262	-0,386	0,058	0,067	-0,141	-0,128	-0,234	-0,170	-0,148	0,070	0,492**	0,140	0,158
**Anaerotruncus**	-0,416	0,110	0,214	0,284*	0,465**	0,075	0,290**	-0,340	-0,091	0,030	0,117	0,003	-0,023	-0,200	-0,042	-0,055	-0,283	-0,081
**Bacteroides**	0,195	0,268	-0,329*	-0,356	-0,038	-0,214	-0,136	-0,142	-0,077	-0,160	-0,351*	-0,039	-0,207	-0,169	-0,095	0,099	-0,404	0,089
**Clostridium sensu stricto 1**	0,579*	-0,375	-0,114	-0,466	-0,518*	-0,245	-0,558*	-0,435	-0,739*	-0,770***	-0,240	-0,366	-0,798***	0,082	-0,136	0,285	0,189	0,032
**Intestinimonas**	-0,088	-0,062	-0,126	-0,126*	-0,208	-0,409	-0,266*	-0,319	-0,573*	-0,292	-0,080	-0,046	-0,302*	-0,049	0,143	-0,231	0,030	-0,064
**Allobaculum**	0,106	-0,338	0,210	-0,059	-0,309	-0,176	-0,247	-0,252	-0,340	-0,354*	-0,455*	0,149	-0,321	-0,203	0,017	0,297	0,071	0,059
**Anaerofustis**	-0,564*	-0,123	0,438**	0,196	0,039	0,032	0,119	-0,166	-0,233	-0,032	0,248	0,350	0,061	0,158	-0,067	-0,601	-0,261	-0,249
**Anaerovorax**	-0,545***	-0,127	0,136	-0,105	0,070	0,074	-0,035	0,037	-0,384	0,083	0,128	0,301	0,002	-0,281	-0,060	-0,678	0,194	-0,008
**Auricoccus-Abyssicoccus**	-0,293	0,281	0,023	0,190	0,302	0,018	0,180	0,234	0,193	0,326	0,412*	0,006	0,455	-0,324	-0,431	0,112	0,136	0,133
**Candidatus Saccharimonas**	-0,302**	-0,107	0,277	0,234	-0,026	0,128	0,185	-0,117	0,163	0,166	0,363*	0,356*	0,273	0,326	-0,295	-0,251	-0,180	-0,329
**Luminal content in Male offspring**																		
**Akkermansia**	0,415	-0,068	-0,273	0,413	-0,169	0,384	0,153	-0,059	0,373	-0,128	-0,186	-0,448	-0,210	0,377*	0,479	0,558*	0,341	0,210
**Anaerostipes**	0,564	-0,419**	-0,281	-0,032	-0,420	0,164	-0,348	0,030	-0,251	-0,030	-0,134	-0,218	-0,203	0,301	0,511	0,458	0,719***	0,077
**Clostridium sensu stricto 1**	0,564	0,070	-0,615	-0,266	-0,735	-0,438*	-0,619	-0,501	-0,103	-0,627	-0,524	-0,102	-0,772	0,117	0,117	0,517	0,144	0,488
**Colidextribacter**	0,158	0,475*	-0,132	0,368	0,389*	0,235	0,428*	0,255	0,239	0,170	0,173	-0,221	0,288	0,055	0,192	-0,203	0,193	0,182
**Defluviitaleaceae UCG-011**	-0,246	0,054	-0,143	-0,264	0,049	-0,471**	-0,194	-0,065	-0,143	-0,012	-0,189	-0,021	-0,090	0,093	0,165	-0,217	-0,325	-0,077
**Eubacterium coprostanoligenes group**	-0,131	0,152	-0,268	-0,662**	0,135	-0,449*	-0,323	0,113	0,114	-0,093	-0,167	0,189	0,112	0,033	-0,220	-0,161	-0,204	-0,106
**Odoribacter**	-0,425***	-0,211	0,259	0,294	0,300	0,718***	0,418**	0,100	0,357	0,532**	0,523**	-0,029	0,361	-0,093	-0,093	-0,224	-0,245	-0,244
**Oscillospira**	0,111	-0,100	0,375	0,468	0,385	0,471	0,468	0,126	-0,007	0,272	0,423	0,026	0,257	0,434**	0,042	0,007	0,229**	-0,113
**UBA1819**	0,006	0,255	0,162	0,294	0,071	0,289	0,344	0,277	-0,164	0,144	0,350**	0,189	0,265	-0,159	-0,104	-0,455	0,239	-0,142
**Allobaculum**	0,424	-0,464**	-0,014	0,105	-0,537	0,350	-0,270	-0,103	0,003	-0,305	-0,170	-0,600**	-0,445	0,301	0,640	0,458	0,330	0,136
**Candidatus Saccharimonas**	-0,504***	-0,366	0,554	0,444	0,031	0,204	0,267	-0,047	0,196	0,167	0,410*	0,345	0,381	0,070	-0,169	-0,257	-0,134	-0,254
**Corynebacterium**	-0,446	0,138	0,086	0,132	0,594***	-0,149	0,274	0,073	0,153	0,272	0,198	0,106	0,428	-0,257	-0,028	-0,242	-0,171	-0,362
**Eubacterium brachy group**	-0,514**	-0,284	0,138	-0,099	-0,089	-0,183	-0,143	0,252	-0,204	0,244	0,103	-0,124	0,056	-0,270	0,243	-0,147	-0,175	-0,135
**Family XIII UCG-001**	-0,067	0,209	-0,115	0,000	-0,306	-0,124	-0,022	0,098	0,258	0,085	-0,042	0,018	0,022	-0,287	0,053	-0,009	-0,392**	0,339
**Lachnospiraceae FCS020 group**	-0,178	-0,100	0,229	0,201	0,135	-0,147	0,025	-0,035	-0,356	-0,107	0,301	-0,125	0,024	0,225	0,211	-0,294	0,178	-0,293
**Monoglobus**	-0,495	0,339	0,415	0,251*	0,386*	-0,065	0,400**	-0,206	-0,046	0,002	0,162	0,746***	0,283	-0,133	-0,094	-0,669*	-0,163	0,039
**Streptococcus**	-0,321	-0,258	0,260	0,166	0,235	-0,068	0,014	-0,175	-0,105	-0,006	0,255**	0,074	0,081	0,035	0,214	-0,287	-0,056	-0,373
**UCG-005**	0,382	-0,091	-0,211	-0,266	-0,693***	-0,201	-0,491	-0,460	-0,154	-0,636**	-0,499	-0,142	-0,636*	0,021	0,329	0,109	-0,045	0,413***
**UCG-009**	-0,378**	-0,289	0,426*	0,632*	0,240	0,490	0,488	0,140	0,311	0,660***	0,636**	0,049	0,475**	0,110	0,033	-0,133	-0,145	-0,364*

Supplementary Table S5: Spearman's rank correlation table of all differentially abundant taxa with social interaction score, neuroinflammatory and morphometric parameters, and TJ markers in male offspring. (*p < 0.10, **p < 0.05, ***p < 0.01).

		Ventral Hippocampus						Dorsal Hippocampus										
**Crypt microbiota in Female offspring**	**SI score**	**IL-6**	**IL-1β**	**TNFα**	**CX3CR1**	**CD68**	**z-score VH**	**IL-6**	**IL-1β**	**TNFα**	**CX3CR1**	**CD68**	**z-score DH**	**Villus lenght**	**Crypt lenght**	**Surface Area**	**Ocln**	**Z0-1**
**Clostridia vadinBB60 group**	0,394	-0,187	-0,031	0,170	0,025	0,198	-0,062	0,109	-0,265	0,143	0,209	-0,140	0,000	0,329	0,161	0,497**	0,378	0,510**
**Clostridium sensu stricto 1**	0,214	0,560	0,227	0,210	0,183	-0,082	0,360	-0,160	0,071	0,204	-0,054	-0,179	-0,057	-0,418	0,479	-0,576	-0,155	-0,307
**Enterorhabdus**	-0,332	-0,307	-0,201	-0,659**	0,035	-0,027	-0,336	0,181	-0,103	-0,058	-0,046	0,003	0,034	-0,119	-0,217	-0,217	0,002	0,310
**Erysipelatoclostridium**	-0,405	-0,369	0,015	-0,137	-0,062	0,134	-0,120	0,283	0,365	0,012	0,277	-0,226	0,179	0,056	-0,573	0,077	0,153	0,072
**Eubacterium coprostanoligenes group**	0,172	0,469***	-0,366	-0,137	0,145	-0,323	-0,020	0,178	-0,021	0,299	-0,063	0,491	0,441	-0,354	0,382	0,014	0,112	0,128
**Muribaculum**	-0,301	0,136***	0,056	-0,417	0,141	0,147	0,055	-0,150	0,304	-0,346	-0,147	-0,259	-0,084	-0,140	0,042	-0,147	-0,207	0,080
**Roseburia**	0,332**	0,122	-0,226	0,205	-0,203	-0,346	-0,084	-0,032	-0,529*	0,311	0,529	0,432**	0,174	-0,400	0,382	-0,373	0,226	0,358
**UCG.009**	0,463	-0,040	-0,373	-0,032	0,388	-0,093	-0,079	-0,319	-0,309	-0,123	0,032	0,203	-0,064	-0,524*	0,182	-0,161	-0,010	0,086
**Candidatus Saccharimonas**	0,370	-0,051	0,009	0,246	0,225	0,081	-0,046	-0,072	0,262	0,490	-0,066	-0,382*	0,281	0,168	0,208	0,647**	0,145	0,191
**Chloroplast**	-0,376	-0,012	0,402	0,314	-0,113	0,073	0,348	0,057	0,308	-0,207	0,038**	-0,364	-0,306	-0,101	-0,083	0,046	0,022	-0,245
**Eubacterium brachy group**	-0,509**	-0,224	-0,011	-0,399**	-0,398	-0,486	-0,363**	0,244	-0,119	0,173	0,174	0,121	0,122	0,145	-0,131	0,000	-0,166	-0,055
**Eubacterium siraeum group**	0,534*	-0,126	-0,476	-0,154	0,092	-0,377*	-0,384	-0,040	-0,225	0,211	0,388	0,305	0,213	-0,480**	-0,044	0,131	0,151	0,449
**Lachnospiraceae.UCG.008**	-0,272	-0,020	0,209	0,014	0,078	-0,023	0,190	-0,097	0,125	-0,066	0,112	-0,361	-0,072	0,029	-0,237	-0,362	-0,120	-0,064
**Monoglobus**	-0,300***	-0,533	-0,175	-0,136	0,051**	0,195	-0,180	0,324	0,205	0,036	-0,257	-0,086	0,012	0,126	-0,399	-0,028	0,155	-0,129
**Paludicola**	-0,439**	-0,377	-0,077	-0,348	-0,332	-0,177	-0,449**	0,143	0,101	-0,428	0,050	0,039	-0,121	0,441	-0,760*	-0,070	-0,220	-0,126
**RF39**	-0,416	-0,446	0,333	0,030	0,138	0,365	0,042	-0,064	0,245	-0,508*	-0,156**	-0,253	-0,515	0,121	-0,216	0,061	-0,044	-0,299
**Syntrophococcus**	0,505	0,222	-0,215	0,206	0,290**	0,186	0,173	-0,169	-0,523*	0,073	-0,013	0,058	-0,195	-0,274	0,707	-0,730*	0,236	0,170
**Tuzzerella**	0,502*	-0,101	-0,336	0,172	-0,128	-0,192	-0,263	-0,092	-0,262	0,031	0,075	0,395***	0,003	-0,450	0,128	-0,670*	0,495	0,327
**UCG.010**	0,376*	0,260	0,019	0,053	0,756*	0,371	0,323	-0,030	-0,309	0,445	0,243	-0,299**	0,032	-0,036	0,374	0,132	-0,200	-0,106
**Luminal content in Female offspring**																		
**Anaerostipes**	-0,237*	-0,072	-0,145	-0,222	-0,322	-0,625*	-0,293	0,091	-0,125	0,343	-0,079	-0,020	0,210	-0,137	-0,112	-0,508	-0,315**	-0,379
**Anaerotruncus**	-0,554***	-0,088	0,035	-0,135	-0,409	-0,416*	-0,204	-0,167	0,275	-0,395*	-0,238	-0,010	-0,164	-0,181	-0,016	-0,308	-0,167	-0,244
**Butyricicoccus**	-0,253*	0,050	0,296*	0,120	-0,255	-0,075	0,029	-0,353**	0,364	-0,238	-0,039	-0,379	-0,229	-0,350	-0,189	-0,364	-0,220	0,009
**Clostridium sensu stricto 1**	0,552	0,785**	0,118	0,476	0,208	0,119	0,433	-0,114	0,033	0,112	0,240	0,178	0,116	-0,098	0,423	-0,116	0,166	0,334
**Clostridia vadinBB60 group**	0,235	-0,449	-0,321	0,081	-0,318	0,104	-0,391	0,275	-0,046	0,042	-0,044	0,199	0,176	0,341	0,038	0,637***	0,450	-0,108
**Erysipelatoclostridium**	-0,480*	-0,143	0,198	0,122	-0,262	0,129	0,141	0,382	0,568*	0,091	0,113	-0,046	0,255	-0,082	-0,335	-0,077	0,247	0,392
**Escherichia.Shigella**	0,170	0,083	-0,186	-0,005	-0,199	0,044	-0,033	0,298	-0,214	0,300	0,064	0,431	0,294	-0,136	0,564	0,182	0,270*	-0,063**
**Incertae Sedis**	-0,367*	0,226	0,505*	0,173	-0,530**	-0,295	0,197	0,075	0,442	0,353	-0,220	-0,176	0,268	0,193	0,438	-0,081	0,043	0,161
**Lachnoclostridium**	-0,129	-0,191	0,030	0,244	-0,290	-0,131	-0,130	-0,087	25,000	-0,265	0,329**	0,110	-0,034	-0,105	-0,573	-0,077	0,186	-0,043
**Lachnospiraceae UCG.006**	-0,257	0,207	0,267*	-0,071	-0,039	-0,513	0,051	-0,339	-0,089	-0,128	-0,145	0,231	-0,138	-0,056	0,137	0,046	-0,189	0,195
**Marvinbryantia**	0,541**	0,397	-0,327	0,225	0,020	-0,108	0,000	0,160	0,121	0,256*	0,315	0,468*	0,421*	-0,183	-0,420	0,009	0,497**	0,018
**Muribaculum**	-0,278	0,083**	0,100	-0,171	0,092	0,248	0,221	0,030	0,247	-0,186	-0,172	-0,329	-0,013	-0,143	0,176	-0,137	-0,292	-0,162
**Candidatus Saccharimonas**	0,498	0,202	-0,233	0,238	0,135	-0,264	-0,106	-0,375**	-0,133	0,179	-0,205	-0,082	-0,136	0,109	0,435*	0,268	0,042	-0,032
**Clostridium methylpentosum group**	-0,153*	0,194	-0,086	-0,111	-0,167	-0,003	0,013	-0,011	0,293	-0,182	-0,319	0,346	0,091	-0,426	0,517	-0,421	0,227	-0,324
**Coriobacteriaceae UCG.002**	-0,763**	0,032	0,406	-0,474	0,149*	-0,085	0,153	-0,179	0,461	-0,321	-0,390	-0,433	-0,248	-0,115	-0,004	-0,074	-0,461	-0,304
**Defluviitaleaceae UCG.011**	-0,550**	-0,561	0,083	-0,269	0,011	0,268	-0,122	0,086	0,143	-0,377	-0,309	-0,272	-0,333	0,368	-0,192	0,335	-0,040	0,077
**Desulfovibrio**	-0,408	-0,260	-0,146	-0,457	-0,327	-0,247	-0,317	0,207	-0,121	-0,147	-0,150	-0,018	0,012	0,350	-0,245	0,056	-0,056	-0,252
**Family XIII UCG.001**	-0,396	0,284*	0,325*	0,302	0,013	-0,198	0,462	0,033	0,217	0,082	0,010	-0,068	0,196	-0,057	0,023	-0,266	-0,097	-0,139
**Haemophilus**	-0,603	-0,244	0,056	-0,214	-0,188	-0,021	-0,062	0,178	0,204	-0,137	0,283	-0,126	0,107	-0,119	-0,763**	-0,407	-0,256	-0,179
**Monoglobus**	-0,198*	0,112	0,134	0,082	-0,212	-0,294	0,122	0,144	0,533	0,334	-0,252	-0,032	0,334	0,246	-0,057	0,185	-0,034	-0,035
**Ruminococcus torques group**	-0,425	-0,046	-0,172	-0,434	0,116	-0,183	-0,252	-0,023	-0,028	-0,168	0,168	-0,028	-0,015	-0,016	-0,591***	-0,237	-0,293	0,226
**UCG.002**	0,660*	0,230	-0,229	0,375	-0,101	0,004	-0,069	-0,304	-0,185	-0,108	-0,134	0,365**	-0,086	-0,167	0,640	0,151	0,266*	0,209

Supplementary Table S6: Spearman's rank correlation table of all differentially abundant taxa with social interaction score, neuroinflammatory and morphometric parameters, and TJ markers in female offspring. (*p < 0.10, **p < 0.05, ***p < 0.01).
